# Genomic adaptations to chemosymbiosis in the deep-sea seep-dwelling tubeworm *Lamellibrachia luymesi*

**DOI:** 10.1186/s12915-019-0713-x

**Published:** 2019-11-18

**Authors:** Yuanning Li, Michael G. Tassia, Damien S. Waits, Viktoria E. Bogantes, Kyle T. David, Kenneth M. Halanych

**Affiliations:** 1 Department of Biological Sciences & Molette Biology Laboratory for Environmental and Climate Change Studies, Auburn University, Auburn, AL 36849 USA; 2 Department of Ecology and Evolutionary Biology, Yale University, 165 Prospect St, New Haven, CT 06511 USA

**Keywords:** Chemosynthetic symbiosis, Cold seep, Comparative genomics, Nutrition mode, Hemoglobins, Toll-like receptor, Aging, Cell cycle

## Abstract

**Background:**

Symbiotic relationships between microbes and their hosts are widespread and diverse, often providing protection or nutrients, and may be either obligate or facultative. However, the genetic mechanisms allowing organisms to maintain host-symbiont associations at the molecular level are still mostly unknown, and in the case of bacterial-animal associations, most genetic studies have focused on adaptations and mechanisms of the bacterial partner. The gutless tubeworms (Siboglinidae, Annelida) are obligate hosts of chemoautotrophic endosymbionts (except for *Osedax* which houses heterotrophic Oceanospirillales), which rely on the sulfide-oxidizing symbionts for nutrition and growth. Whereas several siboglinid endosymbiont genomes have been characterized, genomes of hosts and their adaptations to this symbiosis remain unexplored.

**Results:**

Here, we present and characterize adaptations of the cold seep-dwelling tubeworm *Lamellibrachia luymesi*, one of the longest-lived solitary invertebrates. We sequenced the worm’s ~ 688-Mb haploid genome with an overall completeness of ~ 95% and discovered that *L. luymesi* lacks many genes essential in amino acid biosynthesis, obligating them to products provided by symbionts. Interestingly, the host is known to carry hydrogen sulfide to thiotrophic endosymbionts using hemoglobin. We also found an expansion of hemoglobin B1 genes, many of which possess a free cysteine residue which is hypothesized to function in sulfide binding. Contrary to previous analyses, the sulfide binding mediated by zinc ions is not conserved across tubeworms. Thus, the sulfide-binding mechanisms in sibgolinids need to be further explored, and B1 globins might play a more important role than previously thought. Our comparative analyses also suggest the Toll-like receptor pathway may be essential for tolerance/sensitivity to symbionts and pathogens. Several genes related to the worm’s unique life history which are known to play important roles in apoptosis, cell proliferation, and aging were also identified. Last, molecular clock analyses based on phylogenomic data suggest modern siboglinid diversity originated in 267 mya (± 70 my) support previous hypotheses indicating a Late Mesozoic or Cenozoic origins of approximately 50–126 mya for vestimentiferans.

**Conclusions:**

Here, we elucidate several specific adaptations along various molecular pathways that link phenome to genome to improve understanding of holobiont evolution. Our findings of adaptation in genomic mechanisms to reducing environments likely extend to other chemosynthetic symbiotic systems.

## Background

**Fig. 1:**
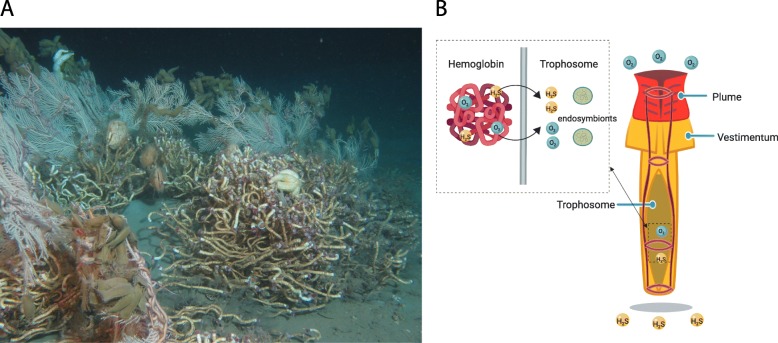
*Lamellibrachia luymesi*. **a** Seep habitat in the Gulf of Mexico. **b** Diagram of adult *L. luymesi* worm to model O_2_ and H_2_S transport to symbionts in trophosome by hemoglobin molecules. The hemoglobin model was created with the help of Biorender (https://biorender.com/)

Recent advances in understanding the dominance of microbes on the planet have placed a new emphasis on elucidating mechanisms that promote microbe-animal symbiosis. Although considerable work has been undertaken on adaptations of microbial genomes to facilitate animal symbiosis (such as corals, termites, humans), examples of how animal host genomes have adapted to symbioses are still limited to a few model systems (e.g., squid-*Vibrio* system and aphid-*Buchnera* system [1–3]). Vestimentiferan tubeworms inhabit some of the Earth’s most extreme environments, such as deep-sea hydrothermal vents and cold seeps, and are obligately dependent on symbiosis for survival. These animals lack a digestive tract and rely on sulfide-oxidizing bacterial symbionts for nutrition and growth. At some seeps, tubeworms, e.g., *Lamellibrachia luymesi* in the Gulf of Mexico, are so abundant that they transform the habitat (Fig. 1a) and thus facilitate biodiversity promoting adaptive radiations and evolutionary novelties [4]. However, previous molecular studies primarily focus on symbionts associated with host lineages living in hydrothermal vents, especially the giant tubeworm *Riftia pachyptila* which is among the best-studied chemoautotrophic symbioses. Given the obligate nature of the symbiosis between tubeworms and their gammaproteobacterial chemoautotrophic endosymbiont, one may reasonably expect adaptations in several cellular mechanisms and pathways (e.g., nutrition, gas exchange, self-defense/self-recognition, control of cell proliferation) to promote efficacy in the symbiotic relationship.

Siboglinid hosts acquire their symbionts from the surrounding environment and store them in a specialized tissue called the trophosome [5]. The chemosynthetic symbionts are known to use a variety of molecules (e.g., H_2_S, O_2_, H_2_) as final electron receptors facilitating a variety of fixation pathways [6]. Primarily, vestimentiferan symbionts use both the reverse TCA cycle (rTCA) and the Calvin cycle for carbon fixation providing a nutrient source for the host [6, 7]. To date, metabolic studies have primarily focused on mechanisms and pathways found in chemosynthetic microbial symbionts, and studies from the host’s perspective are limited.

Another key adaptation contributing to the ability of tubeworms to thrive in chemosynthetic habitats involves hemoglobins (Hbs) that bind oxygen and sulfide simultaneously and reversibly at two different sites [8] (Fig. 1b). Mass spectrometry analyses show siboglinids possess three different extracellular hemoglobins (Hbs): two dissolved in the vascular blood, V1 and V2, and one in the coelomic fluid, C1 [9, 10]. Siboglinid Hbs consist of four heme-containing chains (A1, A2, B1, B2). Sulfur-binding capabilities are hypothesized to be dependent on free cysteine residues at key positions in Hbs, especially in the A2 and B2 chains [8]. V1 Hb can form persulfide groups on its four linker chains (L1–L4), a mechanism that can account for the higher sulfide-binding potential of this Hb [8]. However, sulfide-binding affinity has been suggested to be mediated by the zinc moieties bound to amino acid residues at the interface between pairs of A2 chains in *Riftia* [11]. Thus, which mechanism is primarily responsible for sulfide binding in Siboglinidae is not clear.

Innate immunity is a critical evolutionary driver of maintaining symbiosis [12]. However, little is known about genetic mechanisms relating to immunity and symbiosis. Because tubeworm endosymbionts are housed internally and their establishment process resembles infection [5], tubeworm symbiosis provides a unique opportunity to examine the evolution of immunity functions associated with host-symbiont relationships, such as Toll-like receptors (TLRs) that represent an important mechanism by which the host detects pathogens or commensal microorganisms [13]. Previous studies suggested that several putative cell-signaling and innate immunity genes were more highly expressed in trophosome than plumes in *Ridgeia piscesae*. However, information on extremophile immunity and/or immune tolerance from tubeworm symbiosis is lacking.

Whereas some vent-dwelling vestimentiferans grow rapidly [14], seep-dwelling vestimentiferans have much slower growth rates and are among the most long-lived non-colonial marine invertebrates (up to 250 years) [15]. The cell proliferation activities of the vent-living *Riftia* and seep-dwelling *Lamellibrachia* host cells are, to the best of our knowledge, higher than in any other characterized invertebrates, only being comparable with tumor and wound-healing processes [16]. In contrast with fast-growing *Riftia*, the slow growth of *L. luymesi* maintains a balanced activity of proliferation and apoptosis in the epidermis [16]. Moreover, extremely high levels of apoptosis of host epidermis, muscles, and mesodermal tissue have been observed during the symbiont colonization process in *Riftia* [5]. However, the underlying genetic mechanisms of high rates of cell proliferation and apoptosis, which are directly related to growth and longevity, have not been explored.

The rate at which such adaptations have occurred in this symbiosis is further complicated by the considerable debate concerning the evolutionary timing of siboglinid diversification, owing to conflicting theories of its origins from fossil and molecular age estimates. Siboglinids have been claimed to be as old as 430 mya based on fossil tubes found in the Silurian fossil vent communities [17], but molecular clock analyses suggest a more recent (50–126 mya) origin for vestimentiferans based on mitochondrial COI or 16S gene sequences [18]. Recently, Late Cretaceous *Osedax* fossil traces on reptile falls provided a solid calibration point for the molecular clock of the siboglinids (~ 100 mya) [19]. Moreover, detailed chemical and morphological analyses of tubes from the Figueroa deposits suggested they were made by vestimentiferans, which significantly extends the age of this lineage to a Jurassic origin [20].

To investigate the genomic basis related to tubeworm symbiosis, we report the genomic assembly of the seep-dwelling *Lamellibrachia luymesi* van der Land and Nørrevang 1975. By using comparative genomics, transcriptomic, and proteomic analyses on *L. luymesi*, we provide evidence for the genetic pathways and novel candidate genes which may underlie the extraordinary adaptations of tubeworm symbiosis. In particular, we focus on the mechanisms related to nutrition mode, hemoglobin evolution, immunity function, longevity, and cell cycle to address current hypotheses of controversies. Moreover, we also conduct a detailed molecular clock analysis to discern among the current hypotheses and provide insight on the general timing of such adaptations.

## Results and discussion

### Genome features

Using Illumina paired-end, mate-pair, and 10× genomic sequencing (Additional file 1: Table S1), we assembled the genome of a single *Lamellibrachia luymesi* individual. The haploid genome assembly size is ~ 688 Mb (Additional file 1: Figure S1) with ~ 500× coverage and N50 values of 373 Kb (scaffolds) and 24 Kb (contigs). Although N50 lengths and assembly quality of *L. luymesi* are comparable to those of other annelids (e.g., *Capitella teleta*, *Helobdella robusta*) (Additional file 1: Tables S2, S3), the overall genome completeness measured by BUSCO (~ 95%) is one of the highest among lophotrochozoans (Additional file 1: Table S2). With the support of RNA-Seq data from 3 different tissues (Additional file 1: Table S1), we estimated *L. luymesi* genome contains 38,998 gene models. The genome also exhibits heterozygosity (0.6%) and repetitive content (36.92%) similar to other lophotrochozoans (Additional file 1: Figure S2, Table S4). We found that 94 orthology groups (OGs) appear to have undergone a genomic expansion, and 92 genes appear to be positively selected for specifically in the *L. luymesi* lineage compared to other lophotrochozoan genomes (Additional file 1: Tables S5, S6), and many of them are directly related to the chemosynthetic symbiosis (see below).

### Nutritional adaptations

**Fig. 2:**
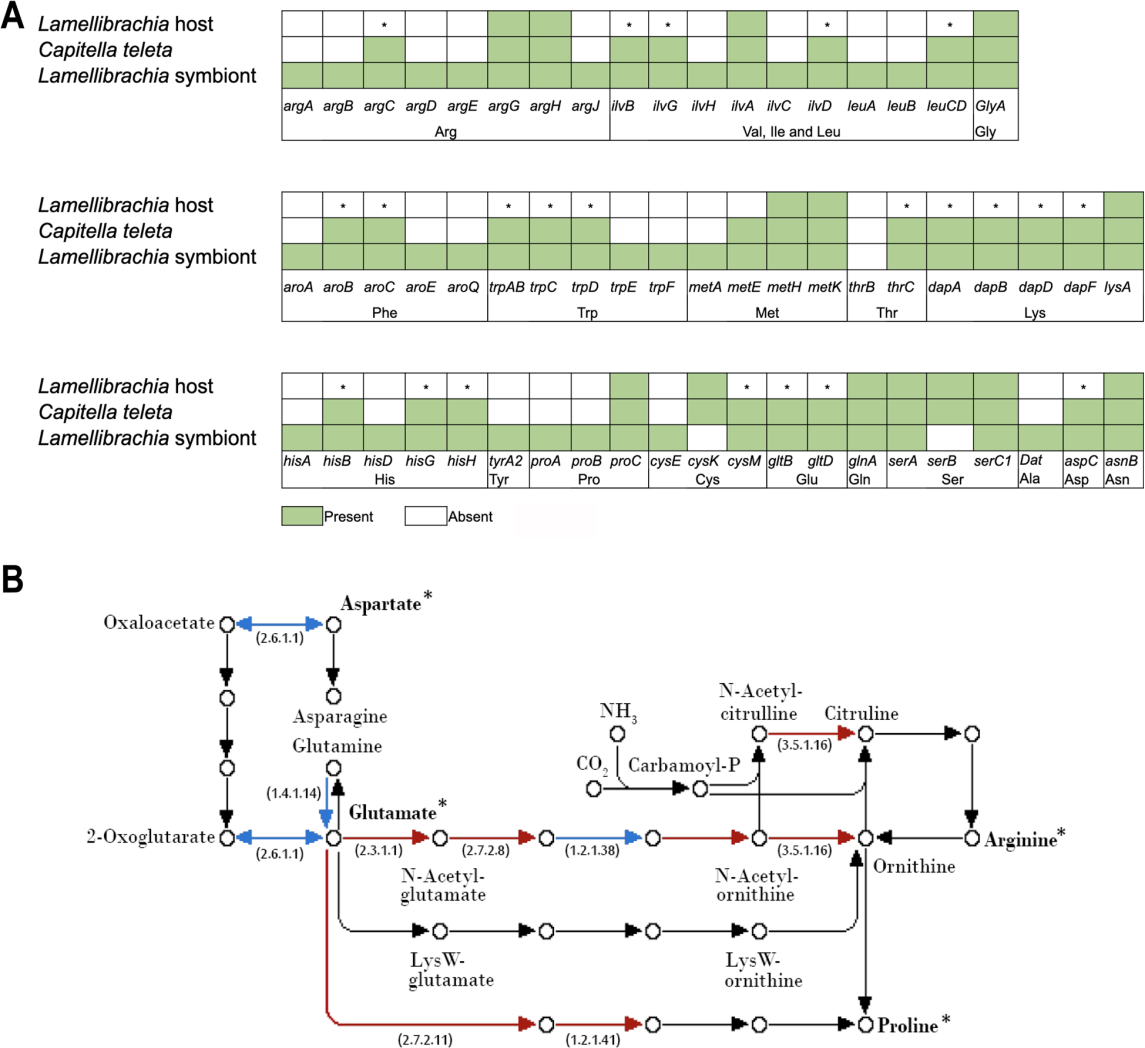
*Lamellibrachia luymesi* lacks amino acid biosynthesis genes. **a** The presence (green) or absence (white boxes) of key genes associated with amino acid biosynthesis in the genomes of *Capitella teleta*, *L. luymesi*, and *L. luymesi* symbionts. The asterisks represent genes present in *C. teleta* and *L. luymesi* gammaproteobacterial symbionts but absent in *L. luymesi*. **b** Aspartate and glutamate biosynthesis pathways as an example of a modified pathway in *L. luymesi*. Blue lines represent key enzymes present in *C. teleta* and *L. luymesi* symbionts but absent in *L. luymesi*. Enzymes only present in *L. luymesi* symbionts are in red boxes. The figure was created with the help of KEGG web server

Only 57 genes associated with amino acid biosynthesis were found in the *L. luymesi* genome, of which 8 were also identified in our proteomic analysis (described below). In contrast, the *Capitella teleta* (Capitellidae, Annelida) genome contains 90 such genes (Fig. 2a; Additional file 2), despite being a less complete and more fragmented genome (Additional file 1: Table S2). These genes were not clustered together in the genomes suggesting that they were probably not missed due to random chance given the completeness of sequencing. Interestingly, the *L. luymesi* symbiont genome contains 110 genes, an essentially complete set for biosynthesis of all 20 proteinogenic amino acids and 11 vitamins/cofactors. Genes found in *C. teleta*’s genome but lacking in *L. luymesi* are involved in the biosynthesis of 13 amino acids (e.g., key enzymes are missing in the Aspartate and Glutamate pathway; Additional file 1: Figure S2B). As amino acids are essential for protein biosynthesis in the host, the lack of many important amino acid synthesis-related genes indicates that the host depends on symbionts for amino acids and cofactors. Moreover, we found a large gene expansion of nutrient uptake ABC transport protein-coding genes in *L. luymesi* compared with other lophotrochozoans (Additional file 1: Table S5). These findings are consistent with previous biochemical analyses which suggest that *Riftia* is also dependent on its bacterial symbiont for the biosynthesis of polyamines that are important for host metabolism and physiology [21].

Obligate bacterial symbionts often lack genes that are commonly found in other free-living bacteria, while retaining only those genes with functions essential to host needs (e.g., in sponges [22], in termites [23]). Although there are known cases of loss in essential gene functions in multicellular eukaryotes, this phenomenon appears to be more frequent in bacterial symbionts [1]. Interestingly, thiotrophic symbionts of the vesicomyid clam *Calyptogena magnifica* [24] and vent mussel *Bathymodiolus azoricus* [25] have been suggested to provide their host with products from amino acid biosynthesis. Moreover, a recent study has suggested that the flatworm *Paracatenula* itself does not store primary energy in host cells; rather, this function is performed by its chemosynthetic symbionts [26]. Although the tubeworms and bivalves under examination in the aforementioned studies live in chemosynthetic environments, the different hosts and bacteria represent disparate genomic backgrounds suggesting that modification and loss of the amino acid biosynthesis pathways may be a convergent adaptation in a variety of chemosynthetic symbioses between bacteria and animals.

In addition to the immediate release of fixed carbon and provision of amino acids by symbionts, we have found proteomic evidence of a second possible nutritional mode whereby the host directly digests symbionts, as shown by the detection of abundant host-derived digestive enzymes in trophosome tissue (Additional file 1: Table S7). Previous observations indicated that symbionts could be digested by *Riftia* [27], but direct evidence and mechanisms related to symbiont digestion were lacking. We identified 15 host proteins related to lysosomal proteases that were both highly expressed and detected as proteins in the trophosome tissue of the host genome, such as saposin and multiple copies of cathepsin (Additional file 1: Table S7). Lysosomes, which contain an array of digestive enzymes, are also thought to play an essential role in symbiont digestion with the chemosynthetic mussel *Bathymodiolus azoricus* [25]. We additionally identified 19 major proteasome components as proteins in the trophosome tissue, indicating a potential role in protein degradation of symbiont digestion (Additional file 1: Table S7). Host lysosomal proteases and proteasome components likely facilitate the degradation of symbionts and may play a role in maintaining appropriate population levels of symbionts within the trophosome.

We also characterized ~ 200 bacterial proteins present in the same trophosome tissue to further understand the host-symbiont interactions. Key enzymatic genes, RubisCO, and ATP citrate lyase (ACL) type II associated with carbon fixation cycles were identified in proteomic analysis from *L. luymesi* (Additional file 1: Table S8). Our results corroborate both rTCA and Calvin cycle, pathways for carbon fixation that might be present in all vestimentiferan endosymbionts [6]. Moreover, consistent with previous analyses [6, 7], several key components related to sulfide and nitrogen metabolic pathways were also identified.

### Hemoglobin evolution

**Fig. 3:**
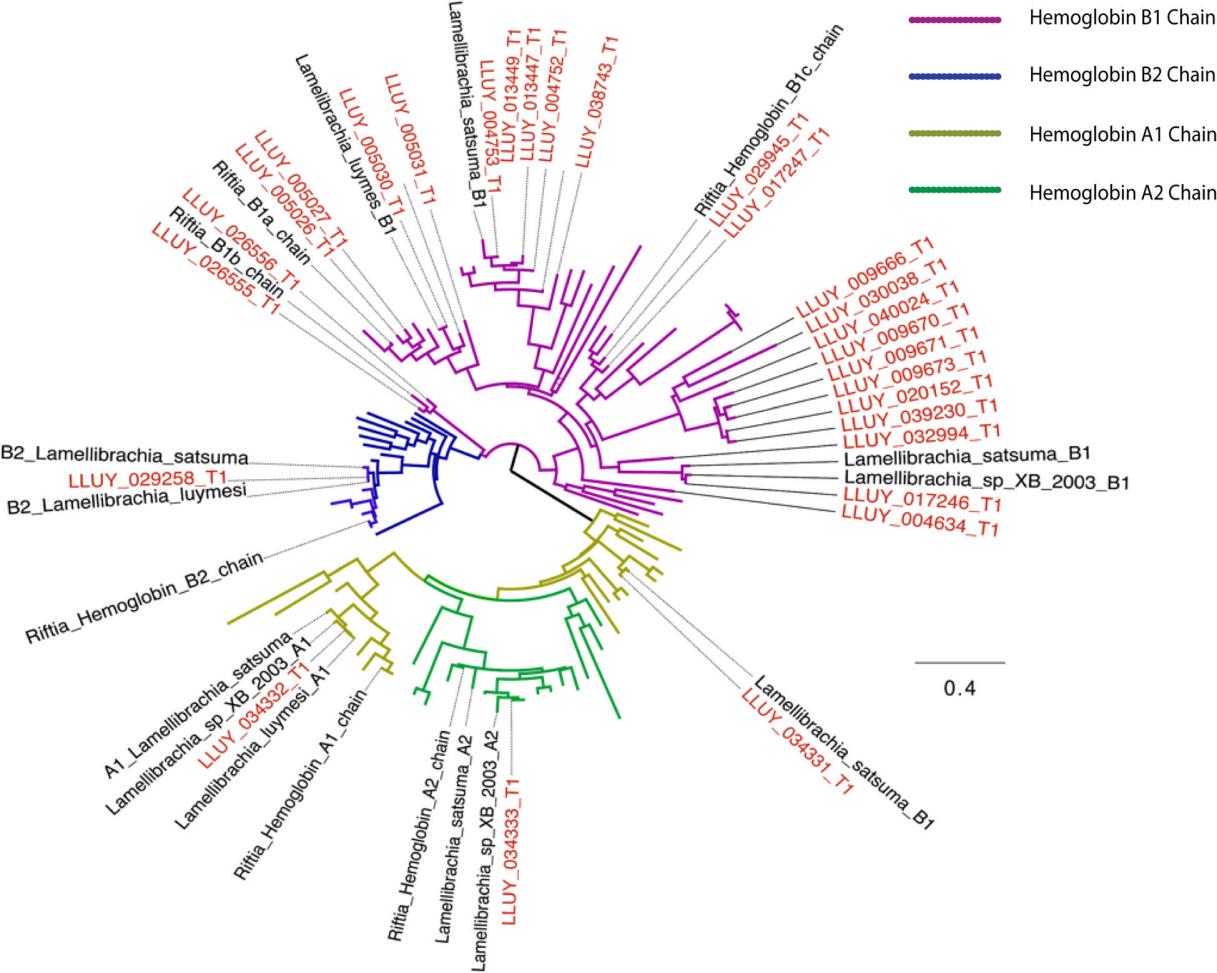
Hemoglobin gene diversity in *Lamellibrachia luymesi*. Gene tree of Hb subunits A1, A2, B1, and B2 from siboglinids and other annelids reconstructed using IQtree with 1000 ultrafast bootstrap. Only siboglinid Hb sequences (from the SwissProt database or this study) were labeled. *L. luymesi* sequences are labeled red, and other annelid HB sequences are not labeled. Accession numbers associated with each sequence are shown in the full tree (Additional file 1: Figure S3)

The mechanisms of Hb sulfide-binding affinity in tubeworm siboglinids are still not clear after 20 years of study [10]. We collected all available Hb sequences from siboglinids and their close relatives and processed them through a phylogenetic framework (Fig. 3, Additional file 1: Figure S3). Importantly, we are able to identify most Hbs and linkers from transcriptomic and proteomic results (Additional file 1: Table S8). Sulfur-binding capabilities are hypothesized to be dependent on free cysteine residues at key positions in Hbs, especially in the A2 and B2 chains [8]. Consistent with previous work [8, 10, 11], in all siboglinids, a single copy of A2 and B2 Hb was identified which possesses a conserved-free cysteine (i.e., cysteine residues not involved in disulfide bridges) at positions 77 and 67, respectively.

Surprisingly, we found a significant expansion of B1 Hbs, 25 copies, in *L. luymesi* whereas most siboglinids and their close relatives only possess 1 copy (Fig. 3), except for *Riftia pachyptila* where 3 B1 Hbs were identified [28]. Noticeably, we found that 8 copies of *L. luymesi* B1 Hb sequences also contains a free cysteine at position 77, the same position as free cysteine in A2 Hbs. More importantly, many B1 Hbs were highly expressed in the trophosome and identified at the protein level (Additional file 1: Table S9). Unlike A2 and B2 globins, B1 globins were long thought to only bind O_2_ and lack the capacity to bind sulfide. Thus, although further analysis is warranted, the presence of the conserved free cysteine might indicate that sulfide-binding capacity also occurred in B1 globins, similar to A2 Hbs. Moreover, the large expansion of hemoglobin B1 genes many of which possess a free cysteine residue suggests that B1 Hbs may play a more important role in tubeworm hemoglobin than previous thought.

Instead of free cysteines mediating H_2_S binding, another hypothesis suggested that zinc moieties bound to amino acid residues at the interface between pairs of A2 chains influence H_2_S binding [11]. The Zn^2+^-binding site contained within the A2 chain is composed of three His residues (B12, B16, and G9) [11]. However, none of these sites is conserved across siboglinids, or even in vestimentiferans (Additional file 1: Figure S5) calling into question the role of the zinc sulfide-binding mechanism for H_2_S transport in siboglinid hosts or at least the role of these particular amino acids in the process.

### Immunity function

**Fig. 4:**
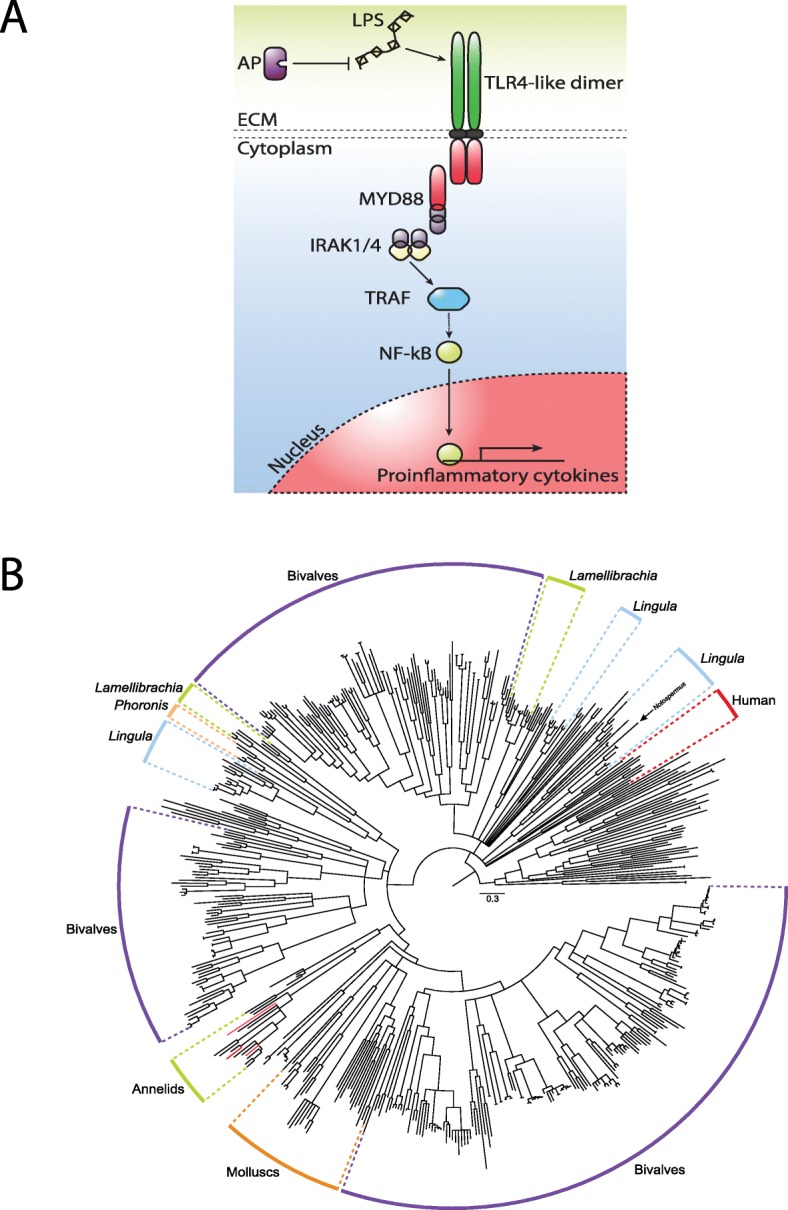
Toll-like receptors (TLRs) in *Lamellibrachia luymesi*. **a** Putative TLR4-like pathway likely essential for immunity and response to symbionts and pathogens. AP, alkaline phosphatase; LPS, lipopolysaccharide. **b** Toll-like receptor gene tree from selected lophotrochozoan genomes and human reconstructed using IQtree with 1000 ultrafast bootstraps. All internal nodes possess ≥ 95% bootstrap support

Immune interactions between hosts and symbionts are a key evolutionary driver that has potential implications for infection by endosymbionts [12], cell cycle, and aging [29]. The genetic machinery and functionality of the immune system in chemosynthetic symbioses have not been extensively characterized. Toll-like receptor (TLR) provides a core cellular and molecular interface between invading pathogens and recognition of host-microbial symbiosis (Fig. 4a). Consistent with previous analyses [30], we found that TLR gene families experienced expansion within lophotrochozoan lineages (Fig. 4b; Additional file 1: Table S10). Within *L. luymesi*, 33 unique TLR proteins were identified compared to 5 in *Capitella telata*, suggesting TLR genes may have additional functions in tubeworms.

A substantial subset of TLR sequences recovered from *L. luymesi* best identify as TLR4 by primary sequence identity and domain structures. In mammals, TLR4 recognizes and binds lipopolysaccharide (LPS; a major cell membrane component of Gram-negative bacteria which include tubeworm symbionts). LPS-bound TLR4 then initiates a signal transduction pathway that activates NF-kB, a transcription factor that promotes the expression of pro-inflammatory cytokines [31] (Fig. 4). *Lamellibrachia luymesi* encodes seven TLR4-like proteins, which is in contrast to the one sequence found in other annelid genomes suggesting a potential for increased sensitivity to Gram-negative bacteria in *L. luymesi*. Interestingly, we found the TAB1 gene (an important component of the TLR4 signaling pathway) is under positive selection (Additional file 1: Table S6). Although the physiological role of TAB genes in the TLR signaling pathway is still unclear [32], it functions as an adaptor protein associated with TAK1 in the TLR4/LPS signaling pathway [33]. We also found genomic expansions of tumor necrosis factor receptors (TNFRs) and TNFR-associated factors (TRAFs) (Additional file 1: Table S5) which play vital roles in the activation and the downstream responses of NF-kB. Thus, these results further support a specialized/expanded role for TLR4-like signaling which is involved in gammaproteobacteria endosymbionts, whereas some other components of the innate immunity (e.g., RIG-1-like receptor signaling pathway which recognizes virus-derived nucleotide present in the cytoplasm) showed no indication of gene expansion.

The initial physical encounter between tubeworms and symbionts occurs in an extracellular mucus secreted by the pyriform glands of newly settled larvae [5]. Within these mucus matrices, symbionts can attach to the host using extracellular components secreted from symbionts, such as LPS. Recognition of lipopolysaccharide (LPS) by TLR4 can result in the induction of signaling cascades that lead to the activation of NF-kB and the production of proinflammatory cytokines [12]. Although the mechanism by which the host distinguishes between symbionts and pathogens in most symbioses is still not clear, alkaline phosphatase has been shown to be involved in the maintenance of homeostasis of commensal bacteria in the squids, mouse, and zebrafish [34]. The commensal bacterially derived LPS signaling via TLR4 yields an upregulation of intestinal alkaline phosphatase and prevents inflammatory responses to resident microbiota. Importantly, we also identified eight copies of alkaline phosphatase, whereas only one copy was found in each of the *Capitella teleta* and *Helobdella robusta* genomes, further supporting a potential mechanism of tolerating Gram-negative bacteria and facilitating symbiotic colonization.

The symbiont’s colonization process induces the activation of innate immune responses and apoptosis of host skin tissue as symbionts travel from host epidermal cells into trophosome [5]. However, the host must coordinate growth, cell proliferation, and cell death precisely during the infection process and tissue homeostasis, as an excessive reaction could lead to deleterious systemic hyperinflammation [16, 29]. Interestingly, the NLRP gene family, which plays a key role in an innate immunity recognition of infectious pathogens and regulates inflammatory caspases [35], showed a large expansion relative to other lophotrochozoans (Additional file 1: Table S5). Moreover, we found a large expansion of Sushi domain-containing genes that are potentially involved in recognition and adhesion between hosts and symbionts (Additional file 1: Table S5).

The genetic components underlying the innate immunity in *L. luymesi* have been highly modulated when compared with its non-symbiotic annelid relatives, in favor of the hypothesis that innate immunity is involved in endosymbiont acquisition and selective tolerance. A TLR4-like signaling pathway may be central for host immunity and in distinguishing between symbionts and pathogens (Fig. 4a).

### Cell cycle and aging

*Lamellibrachia luymesi* proteomic and genomic data show that several apoptosis systems, including TNFR, TRAF, and two caspase gene families (caspase 3 and caspase 7), are expanded relative to other lophotrochozoans. As the key player of apoptosis, caspases initiate the transduction of apoptosis signal by activating the members of TNFR and their DEATH receptor [36]. A previous study has suggested caspase 3 also plays a central role in apoptosis of gill tissue in *Bathymodiolus* mussels [37]. We also found the alpha spectrin gene showed evidence of positive selection in *L. luymesi* genome. Spectrin is one of the major components responsible for maintaining cytoskeletal integrity of the cell and is targeted by caspase 3-mediated cleavage and initiates dissolution of the cytoskeleton during apoptosis [38]. Thus, caspase 3-mediated apoptosis might have important implications in high apoptosis rates in *L. luymesi*. Moreover, we found other genes showing evidence of positive selection including BECN and APOPT1, whose functions have been linked to apoptosis and autophagy [39].

In terms of cell proliferation, four copies of SMAD4 genes were found in the *L. luymesi* genome, whereas only one copy was found in each of the *C. teleta* and *Helobdella robusta* genomes. Of these, one copy of the SMAD4 gene is under positive selection (Additional file 1: Table S6). SMAD4 is a key component in the TGF-β signaling pathway and can also function as a tumor suppressor protein in animals [40]. In some animals, loss of expression of SMAD4 can lead to resistance to growth inhibition and uncontrolled proliferation, such as cancer cells [41]. Thus, based on the expansion and signature of positive selection in SMAD4 genes, we hypothesize that SMAD4 genes may have been an important factor of ultra-high cell proliferation rates in vestimentiferans.

Seep-living vestimentiferans are long-lived, and in addition to innate immunity, our analyses highlighted families that may play a direct role in aging. Superoxide dismutases (SODs) have an important functional role to protect cells against oxidative damage induced by metabolism and are implicated in aging. We found genomic expansions of CuZn superoxide dismutase (SOD1) genes and Mn superoxide dismutase (SOD2) in *L. luymesi*’s genome compared to other lophotrochozoans (Additional file 1: Figure S6). Most lophotrochozoan genomes contain one or two copies of SOD1 and SOD2, but *L. luymesi* has five copies of each gene (Additional file 1: Figure S6). Three of five SOD2 genes were recovered in transcriptomic and proteomic data (Additional file 1: Table S7). Previous studies suggested that overexpression of SOD1 or SOD2 could significantly extend lifespan in mammals, fruit flies, and *Caenorhabditis. elegans* [42]. Moreover, previous studies also suggested that the SOD gene product may help symbionts overcome host cellular immune responses [43]. Thus, although further work is warranted, SODs from both bacteria and tubeworms may play a central role for overcoming oxidative damage and could be essential for extreme longevity for seep-living vestimentiferans.

### Timing of tubeworm diversification

**Fig. 5:**
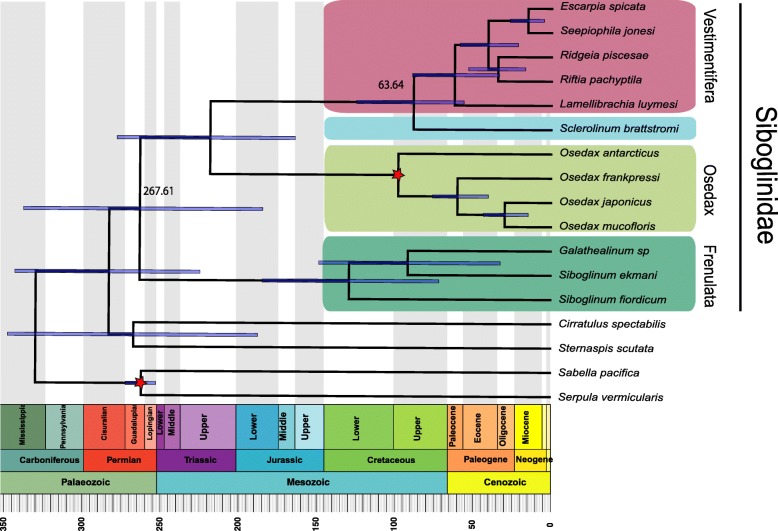
Time-calibrated phylogeny of Siboglinidae inferred with BEAST2 based on 191 OGs in units of millions of years. 95% confidence intervals of divergence time estimate and displayed on nodes. Stars represent nodes that were used as fossil calibration points during age estimation

Molecular clock analyses based on a phylogenomic dataset of 191 genes suggest modern siboglinid diversity originated 267 mya (± 70 my), and support previous hypotheses of a vestimentiferan origin of approximately 50–126 mya in the Late Mesozoic or Cenozoic (Fig. 5). Previous analyses of diversification time were based solely on COI sequences and limited taxon sampling (mainly vestimentiferans), and the time of tubeworm origin is solely estimated by mitochondrial substitution rates [18, 44]. However, mitochondrial genes of vestimentiferans may have experienced a “slow-down” in the rate of nucleotide substitution relative to other siboglinid lineages [44, 45].

Vestimentiferans appear to have originated during the Cenozoic (63.6 mya ± 12) (Fig. 5), contrary to recent analyses that indicated that Jurassic tubes from Figueroa deposits were likely to have been made by vestimentiferans [46]. This adds to the growing evidence that the Cenozoic was a key period for the radiation of most dominant invertebrate taxa now occupying in deep-sea chemosynthetic communities [46, 47]. By comparison, the common siboglinid ancestor dates as far back as the Permian (267 mya ± 70). When comparing the fossil calibrated and uncalibrated trees, we found the branching order is the exact same and the length of the branch of the key nodes (nodal heights) are largely consistent suggesting the choice of calibration points was appropriate (Additional file 1: Figure S9).

## Conclusions

Symbioses between bacteria and animals are ubiquitous, and ecosystems (e.g., seeps, hydrothermal vents, and organic falls) driven by chemoautotrophy have received considerable attention because of the non-photosynthetic energy source. Despite this interest, the underlying genomic machinery that led to the evolutionary success of these symbiotic systems is poorly understood, especially for hosts. By characterizing the genome of the seep-dwelling tubeworm *Lamellibrachia luymesi*, we provide genetic evidence of how animals adapted to extreme environments and maintain chemosynthetic symbiosis. Analyses show that *L. luymesi* has lost key genes for amino acid biosynthesis making it obligately dependent on endosymbionts. Additionally, expansions have occurred in a number of gene families (e.g., TLRs, SODs, hemoglobins) that have been implicated in bacterial symbiosis. Evolutionarily, increasing the number of paralogs provides an opportunity for neofunctionalization or subfunctionalization, allowing more refined gene-gene interactions to promote symbiotic efficacy. This balance of gene family expansion and gene loss may be a hallmark of how genomic machinery adapts and develops interdependence across a variety of bacterial-animal symbioses.

## Methods

### Organismal collection

*Lamellibrachia luymesi* was collected from seeps in the Mississippi Canyon in the Gulf of Mexico (N 28° 11.58′, W 89° 47.94′, 754 m depth), using the *R/V Seward Johnson* and *Johnson Sea Link* in October 2009. Samples were frozen at − 80 °C following recovery.

### Genome sequencing and assembly

Using vestimentum tissue of one individual, high-molecular weight genomic DNA was extracted using the DNeasy Blood & Tissue Kit (Qiagen). Four TruSeq paired-end and two Nextera mate-pair genomic DNA libraries were generated and sequenced by The Genomic Services Lab at the Hudson Alpha Institute for Biotechnology in Huntsville, AL, on an Illumina HiSeq platform (Additional file 1: Table S1). Additionally, HudsonAlpha constructed and sequenced a Chromium 10× sequencing library (10× genomics) from the same individual on an Illumina HiSeqX platform.

Our genome assembly workflow is shown in Additional file 1: Figure S7, and the commands for bioinformatic pipelines used herein can be found in Additional file 3. Further details on the commands used for the genome assembly and all the analyses described below are available in Additional file 3. Paired-end and 10× raw reads were checked with FastQC v0.11.5 [48] and quality filtered (*Q* score > 30) with Trimmomatic v0.36 [49]. Genome size, level of heterozygosity, and repeat content were determined using *k*mer histograms generated from the paired-end libraries in Jellyfish v2.2.3 [50] and GenomeScope [51] (Additional file 1: Figure S1). Mate-pair reads were trimmed and sorted using NxTrim v0.3.1 [52], and only “mp” (true mate-pair reads) and “unknown” (mostly large insert size reads) reads were used for downstream scaffolding analysis.

Given high heterozygosity in non-model species, all reads were assembled using Platanus v1.2.4 [53] with a *k*mer size of 32. Scaffolding was conducted by mapping PE and MP reads to Platanus contigs using SSPACE v3.0 [54]. Gaps in the scaffolds were filled with GapCloser v1.12 [55], and redundant allele scaffolds were removed using Redundans v0.13c (default settings [56]). Genome assembly quality was assessed with QUAST v4.5 [57] and genome completeness with BUSCO v3 [58] using the Metazoa_odb9 database (978 Busco genes). We also attempted to assemble the genome using 10× data in Supernova 1.2.0 [59], but the genome quality and completeness were inferior to the Platanus assembly (Additional file 1: Figure S7) and thus ignored in the downstream analysis. Short genomic assemblies (< 500 bp) which resulted from repetitive genomic regions and potential contamination from hosts were excluded from the assembly. Finally, to remove putative contaminants from symbionts, BLAST [60] was performed on genome assemblies using the *Lamellibrachia* symbiont genome from the previous study [6] as the bait sequence using an *e* value cutoff of 1e−5, and no BLAST hit was identified.

### Transcriptome assembly and analysis

Total RNA was extracted by TRIzol (Thermo Fisher Scientific) from the plume, vestimentum, and trunk/trophosome tissue of the same *L. luymesi* and purified using the RNeasy kit (Qiagen) with on-column DNase digestion. cDNA library construction and RNA-Seq was carried out by HudsonAlpha using an Illumina HiSeq 2000 platform. After the raw reads were checked with FastQC v0.11.5 and quality filtered (*Q* score > 30) with Trimmomatic v0.36., transcripts were assembled in Trinity v2.4.0 [61] with default settings and a *k*mer of 31. Transcript isoforms with high similarity (≥ 95%) were removed with CD-HIT-EST v4.7 [62]. Transcripts were verified and abundance estimated by read mapping with Bowtie v2.2.9 [63] and RSEM v1.2.26 ([64] back to the transcript assembly.

### Genome annotation

Gene models were constructed following the Funannotate pipeline 1.3.0 (Additional file 1: Figure S8) using information from the genome assembly, transcriptome assembly, and SwissProt/Uniprot. For genome data, repetitive regions were identified using RepeatModeler v1.0.8 [65] and soft-masked using RepeatMasker v4.0.6 [66]. For each transposable element (TE) superfamily, relative ages of different copies were estimated by calculating Kimura distances assuming that most mutations are neutral. RNA-Seq data were combined into a single de novo assembly with Trinity, and a spliced alignment was indexed against the genome assembly with HISAT 2.1.0 [67]. The PASA pipeline v2.3.3 [68] was used to identify high-quality gene models that were used to train the ab initio gene predictor in AUGUSTUS v3.3 [69] and GenMark. Additionally, SwissProt protein data was aligned to the genome assembly using Exonerate [70] and *L. luymesi* transcripts aligned using Minimap2 v2.1 [71]. tRNA genes were identified with tRNAscan-SE v1.3.1 [72]. Finally, EvidenceModeler 1.1.0 [73] was used to combine all evidence of gene prediction from protein alignments, transcript alignments, and ab initio predictions to construct high-quality consensus gene models. Functional annotations of predicted gene models were performed using curated databases: KEGG Orthology was assigned using the KEGG Automatic Annotation Server [74], domain structure by InterProScan [75], and protein identity with the SwissProt database. Secreted proteins were predicted using SignalP [76] and Phobius [77] in InterProScan.

### Proteomics characterization

Proteomic analysis of *Lamellibrachia luymesi* trunk/trophosome tissue was performed by Proteomics & Metabolomics Facility at Colorado State University. Here, we restate the protocol provided by the Colorado State University. Fifty micrograms total protein was aliquoted from each sample and processed for in-solution trypsin digestion as previously described [78]. A total of 0.5 μg of peptides were then purified and concentrated using an online enrichment column (Waters Symmetry Trap C18 100 Å, 5 μm, 180 μm ID × 20 mm column). Subsequent chromatographic separation was performed on a reverse phase nanospray column (Waters, Peptide BEH C18; 1.7 μm, 75 μm ID × 150 mm column, 45 °C) using a 90-min gradient: 5–30% buffer B over 85 min followed by 30–45% B over 5 min (0.1% formic acid in ACN) at a flow rate of 350 nL/min. Peptides were eluted directly into the mass spectrometer (Orbitrap Velos Pro, Thermo Scientific) equipped with a Nanospray Flex ion source (Thermo Scientific), and spectra were collected over a *m*/*z* range of 400–2000 under positive mode ionization. Ions with charge state + 2 or + 3 were accepted for MS/MS using a dynamic exclusion limit of 2 MS/MS spectra of a given *m*/*z* value for 30 s (exclusion duration of 90 s). The instrument was operated in FT mode for MS detection (resolution of 60,000) and ion trap mode for MS/MS detection with a normalized collision energy set to 35%. Compound lists of the resulting spectra were generated using Xcalibur 3.0 software (Thermo Scientific) with a S/N threshold of 1.5 and 1 scan/group.

Tandem mass spectra were extracted, charge state deconvoluted, and deisotoped by ProteoWizard MsConvert v3.0. Spectra from all samples were searched using Mascot (Matrix Science, London, UK; version 2.6.0) against the gene models of *Lamellibrachia* host and symbiont genomes (derived from [6]) assuming the digestion enzyme trypsin. Mascot was searched with a fragment ion mass tolerance of 0.80 Da and a parent ion tolerance of 20 PPM. Oxidation of methionine and carbamidomethyl of cysteine were specified in Mascot as variable modifications. Search results from all samples were imported and combined using the probabilistic protein identification algorithms [79] implemented in the Scaffold software (version Scaffold_4.8.4, Proteome Software, Inc., Portland, OR) [80]. Protein identifications were accepted if they could be established at greater than 99.0% probability and contained at least one identified peptide. Protein probabilities were assigned by the ProteinProphet algorithm [81]. Proteins that contained similar peptides and could not be differentiated based on MS/MS analysis alone were grouped to satisfy the principles of parsimony.

### Gene family analysis and positive selection

Following all-to-all Diamond v1.0 [82] BLASTP searches against 22 selected lophotrochozoan proteomes (Additional file 1: Table S3), orthology groups (OGs) were identified using Orthofinder with a default inflation parameter (*I* = 1.5). Gene ontology annotation used PANTHER v13.1 [83] with the PANTHER HMM scoring tool (pantherScore2.pl). Gene family expansion and contraction were estimated using CAFÉ v2.1 [84]. For each gene family, CAFÉ generated a family-wide *P* value, with a significant *P* value indicating a possible gene family expansion or contraction event. Significantly expanded gene families (*p* < 0.05) were then identified by InterProscan.

Positive selection was calculated using the adaptive branch site-random effects likelihood (aBS-REL) model implemented in HyPhy v. 2.3.9 [85, 86] using the 6000 single-copy genes identified from Orthofinder. Each gene was aligned by codon positions using TranslaterX [87]. Each gene was first tested for positive selection only on the *L. luymesi* branch. Genes under positive selection were tested at all nodes and branches in the phylogeny. *P* values were corrected for multiple testing (using the Holm-Bonferroni correction).

### Manual annotation of gene families of potential interest

We manually annotated particular gene families of interest including hemoglobin gene families, genes related to amino acid synthesize, immunity function, and longevity. Hbs and linker sequences (Additional file 1: Figure S4) of interest were obtained from *L. luymesi* genome and assembled siboglinid transcriptomes derived from previous studies [88, 89] via Diamond BLASTP (*e* value cutoff 1e−5) with *Riftia* Hbs and linker sequences (downloaded from SwissProt Database) serving as bait. Sequences with best hits to target proteins were annotated for protein domain architecture using the Pfam databases included in InterProscan. After manual removal of redundant and incorrect sequences (e.g., sequences are too short or lack of globin domain), we used MAFFT 7.2.15 [90] to align Hb amino acid sequences. Maximum likelihood analyses were performed in IQTree v1.5 [91] under the best-fitting models for associated partition schemes determined by Modelfinder implemented in IQTree with ultrafast bootstrapping of 1000 replicates.

Discovery of SODs and immunity-related genes largely follows the same workflow as used for Hbs using lophotrochozoan SOD genes in the SwissProt database as baits. For immunity genes, targeted genes were additionally processed through the Extract_Homologs2 script used in [92]. We examined major signaling components of the TLR signaling pathway, as well as RLRs, NFkB-associated proteins, and interferon regulatory factors. We only included identification of TLR and RIGs signaling components in the manuscript as other immunity-related genes did not clearly reveal any evolutionary patterns of interest across lophotrochozoans (Additional file 1: Table S10). Importantly, the Extract_Homologs2 script identifies unique protein sequences within an amino acid dataset that fall within user-defined domain architecture criteria (Additional file 1: Table S12). Due to this stringency, the pipeline only identifies the complement of unique proteins for any target family encoded in a genome. Full amino acid sequences for TLRs were placed in a phylogenetic context using the bioinformatic workflow delineated above for Hbs.

Searches for genes related to amino acid synthesis from *Lamellibrachia*, *Lamellibrachia* symbionts, and *Capitella teleta* genomes were performed using the KEGG2 KAAS genome annotation web server and then visualized by the KEGG Mapper Reconstruct Pathway. Additionally, we supplemented the KEGG annotations with BLASTP searches to the SwissProt database.

### Phylogenomics and molecular clock analysis

Analysis of siboglinid phylogeny was conducted utilizing publically available transcriptomic datasets (*n* = 16) in conjunction with our newly generated *Lamellibrachia* proteome (Additional file 1: Table S11). Sequence assembly, annotation, homology evaluation, gene tree construction, parsing of genes trees to OGs, and supermatrix construction were all conducted with Agalma [93]. The reconstructed phylogeny was consistent with previous studies [45, 88] but included 2 additional *Osedax* taxa. The final supermatrix dataset contains 191 single-copy orthologs.

For the molecular clock analysis, a relaxed molecular clock with a log-normal distribution and a Yule tree model was used in BEAST2 v2.5.1 [94] (Fig. 5). Multiple calibration points were used. One calibration was placed on the node representing the most recent common ancestor (MRCA) of *Osedax* using a normal distribution with a mean of 100 mya and a standard deviation of 10 following the findings of *Osedax* burrows in fossils [19]. Another calibration was placed on the node of MRCA of Serpulida and Sabellida using a normal distribution with a mean of 267 mya [95]. Molecular clock analyses with BEAST2 consisted of two independent runs with 10 million MCMC generations sampled every 1000 generations. Convergence was confirmed by comparing trace plots in Tracer ensuring the effective sample size of each parameter was greater than 100 and that stationarity appeared to have been achieved. Log and tree files were combined using Logcombiner. A maximum clade credibility tree with mean heights was calculated using TreeAnnotater. The resulting time-calibrated tree was plotted using an R package, phyloch, strap [96], and OutbreakTools [97]. Bayesian inference using BEAST2 resulted in identical branching patterns as previous studies [45, 88]. Moreover, we also conducted the molecular clock analysis without fossil calibration points to test how the branch lengths vary between fossil calibrated and uncalibrated trees with the same settings above using BEAST2 (Additional file 1: Figure S9).

## Supplementary information

**Additional file 1: Figure S1.** Estimation of genome size. **Figure S2.** Transposable elements in the *Lamellibrachia* genome. **Figure S3.** Siboglinid hemoglobin maximum-likelihood tree. **Figure S4.** Hemoglobin gene diversity. **Figure S5.** Partial alignment of sampled siboglinid HB. **Figure S6.** Lophotrochozoan SOD maximum-likelihood tree. **Figure S7.** Workflow of *Lamellibrachia luymesi* genome assembly. **Figure S8.** Workflow of *Lamellibrachia* genome annotation pipeline. **Figure S9.** Uncalibrated phylogeny of Siboglinidae inferred with BEAST2. **Table S1.** Sequencing information of *Lamellibrachia* genome. **Table S2.** Genome assembly and BUSCO statistics. **Table S3.** Proteomics and genome assemblies. **Table S4.** Repetitive element. **Table S5.** PANTHER gene family annotation. **Table S6.** Genes under positive selection. **Table S7.** Key genes of host genes identified from proteomic. **Table S8.** Key genes of symbiont genes genes identified from proteomic. **Table S9.** *Lamellibrachia* Hb sequences. **Table S10.** Number of unique TLR proteins. **Table S11.** Taxon sampling and source of data used in molecular clock analyses. **Table S12.** Domain requirements for identifying components of TLR pathway.

**Additional file 2: Dataset 1.** Genes identified in annelid genomes related to amino acid synthesis pathways.

**Additional file 3:** Bioinformatic command lines used in this study.

## Data Availability

Raw reads, assembled genome sequences, and annotation are accessible from NCBI under BioProject numbers PRJNA516467 [98], Sequence Read Archive accession numbers SRR8519110–SRR8519119, and Whole Genome Shotgun project number SDWI00000000. The genome annotations, proteomic results, and data for the analyses are available from the GitHub Repository at https://github.com/yuanning-li/Lamellibrachia-genome [99]. Further details on the commands used for all the analyses are available in Additional file 3 [100].
