# Supplementary material for: Genomic adaptations to chemosymbiosis in the deep-sea seep-dwelling tubeworm Lamellibrachia luymesi
Source: BMC Biol. 2019 Nov 18;17:91. doi: 10.1186/s12915-019-0713-x (PMC6862839; doi:10.1186/s12915-019-0713-x)
Supplement: Supplementary file 1 — Additional file 1: Figure S1. Estimation of genome size. Figure S2. Transposable elements in the Lamellibrachia genome. Figure S3. Siboglinid hemoglobin maximum-likelihood tree. Figure S4. Hemoglobin gene diversity. Figure S5. Partial alignment of sampled siboglinid HB. Figure S6. Lophotrochozoan SOD maximum-likelihood tree. Figure S7. Workflow of Lamellibrachia luymesi genome assembly. Figure S8. Workflow of Lamellibrachia genome annotation pipeline. Figure S9. Uncalibrated phylogeny of Siboglinidae inferred with BEAST2. Table S1. Sequencing information of Lamellibrachia genome. Table S2. Genome assembly and BUSCO statistics. Table S3. Proteomics and genome assemblies. Table S4. Repetitive element. Table S5. PANTHER gene family annotation. Table S6. Genes under positive selection. Table S7. Key genes of host genes identified from proteomic. Table S8. Key genes of symbiont genes genes identified from proteomic. Table S9. Lamellibrachia Hb sequences. Table S10. Number of unique TLR proteins. Table S11. Taxon sampling and source of data used in molecular clock analyses. Table S12. Domain requirements for identifying components of TLR pathway. [file 12915_2019_713_MOESM1_ESM.pdf]

Additional file 1

Genomic adaptations to chemosymbiosis in the deep-sea seep-dwelling tubeworm *Lamellibrachia luymesi*

Yuanning Li,*, Michael G. Tassia, Damien S. Waits, Viktoria E. Bogantes, Kyle T. David, Kenneth M. Halanych

Corresponding authors: Yuanning Li, Kenneth M. Halanych
Email: yzl0084@auburn.edu ken@auburn.edu

Additional file 1 includes:

Supplementary Figs. S1 to S8
Supplementary Tables S1 to S12

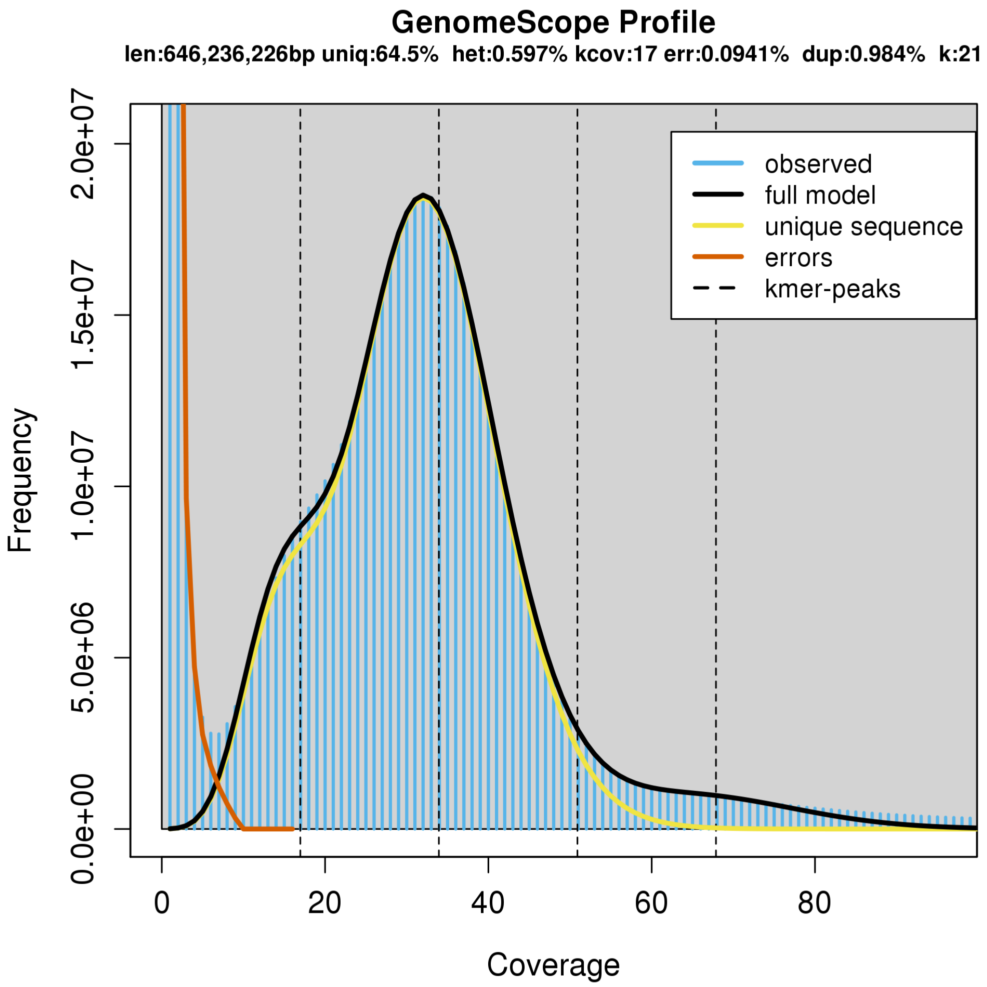

Figure S1. Estimation of genome size, repetitive content and level of heterozygosity from 100 million Illumina paired-end reads.

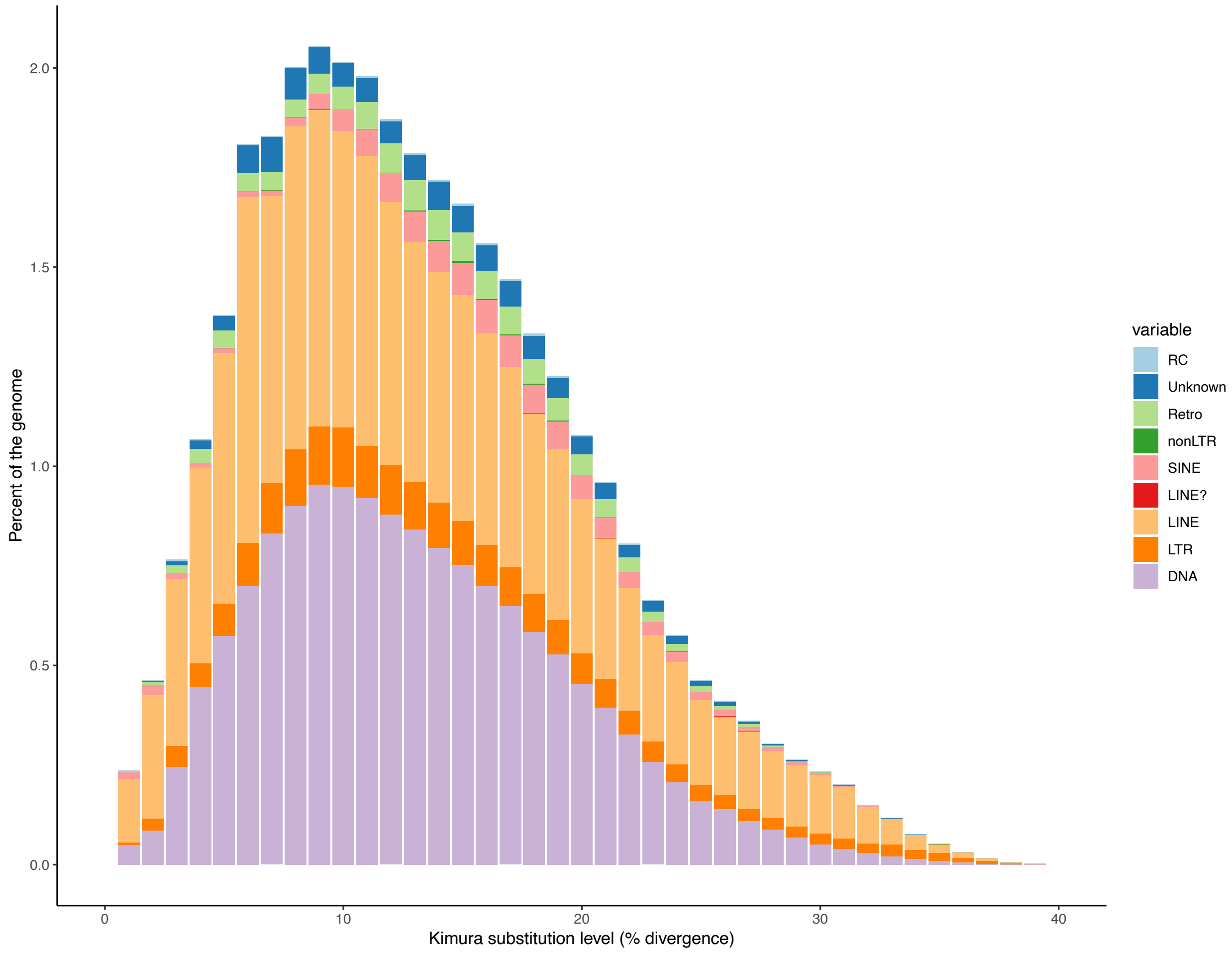

Figure S2. History from major superfamilies of transposable elements in the *Lamellibrachia* genome**.** Kiruma distances are arranged from value 0 representing recent TE copies to 40 for ancient TE insertions.

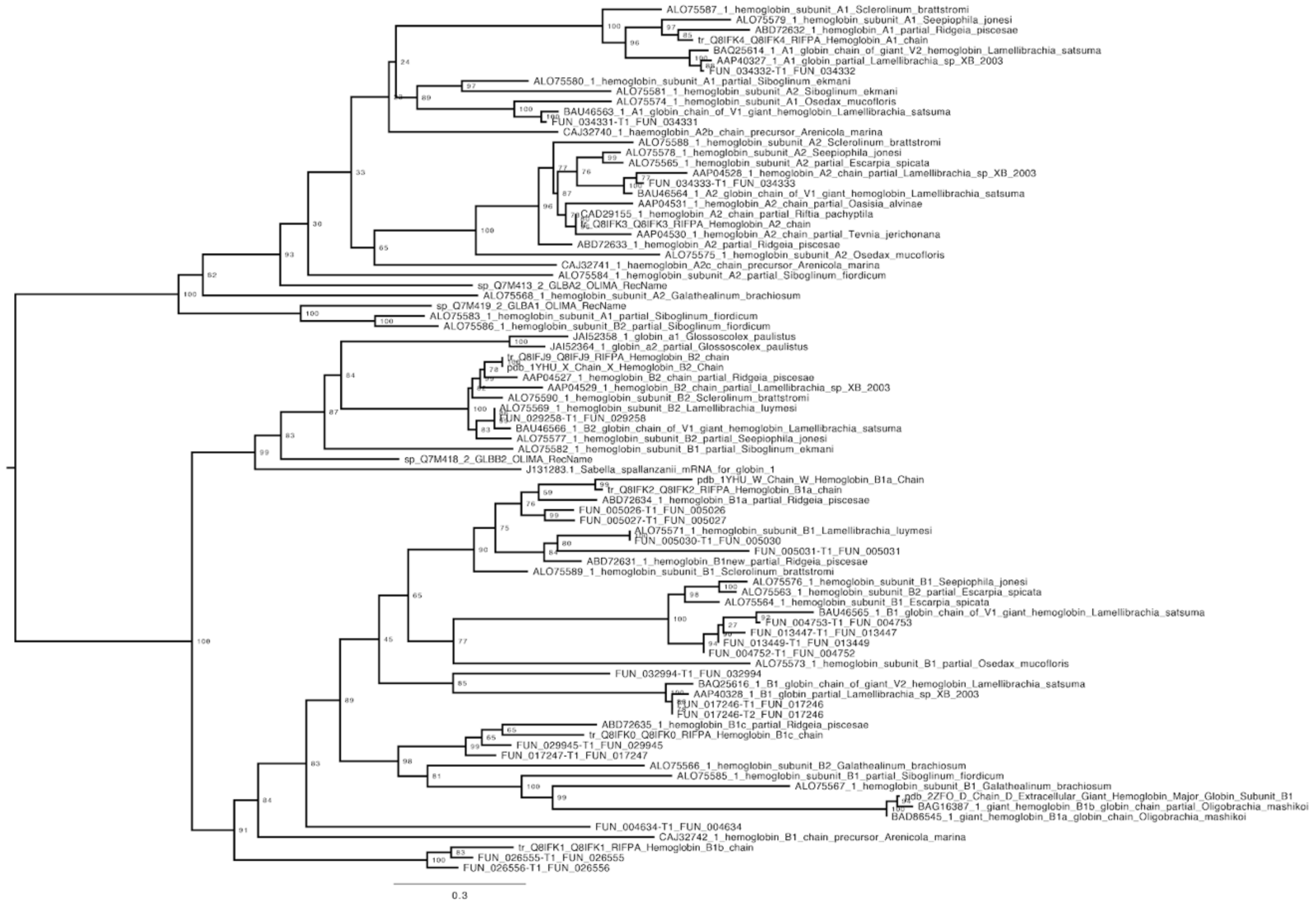

Figure S3. Siboglinid hemoglobin maximum-likelihood tree reconstructed with IQtree with midpoint rooting with 1000 ultrafast bootstraps using LG model. Bootstrap support values are shown at the relevant node. GenBank accession numbers are listed on the terminal nodes.

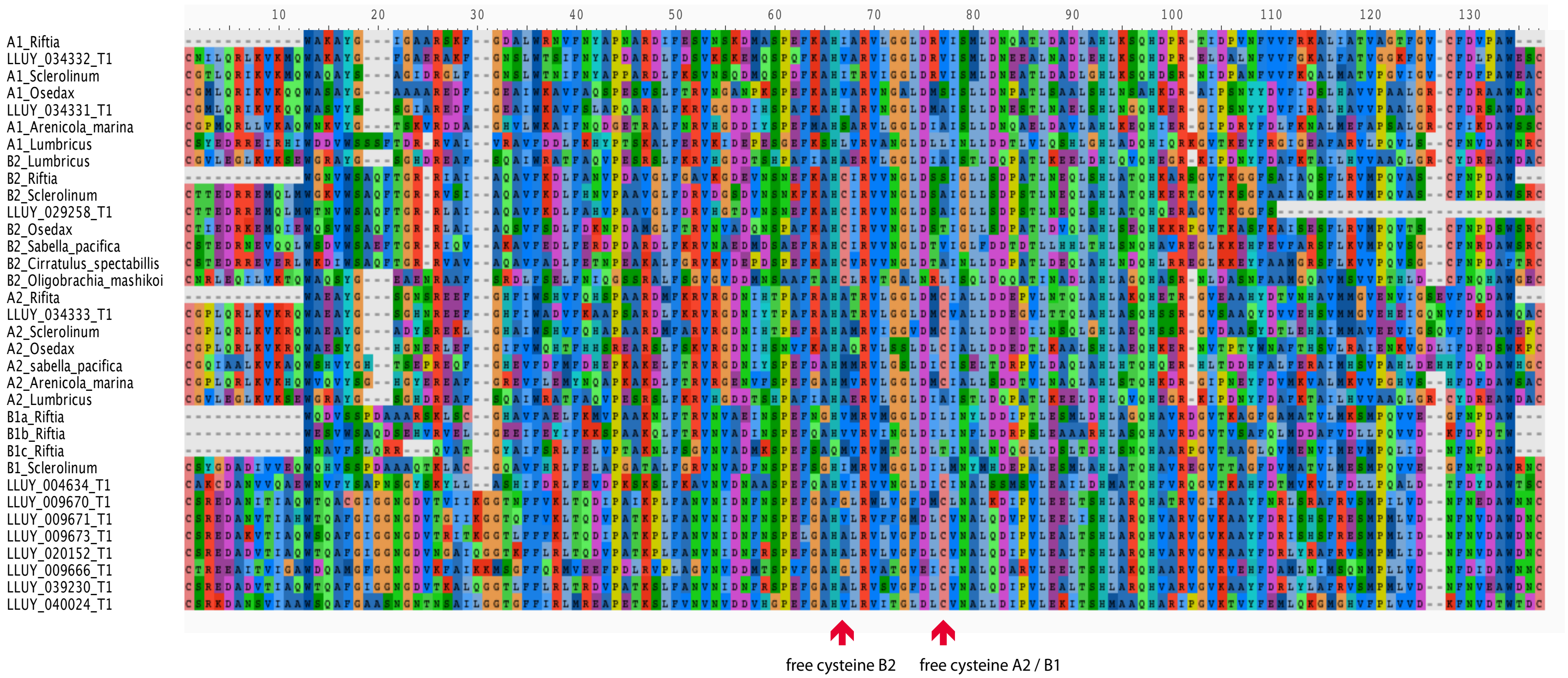

Figure S4. Hemoglobin gene diversity in *Lamellibrachia luymesi*. (Partial alignment of sampled siboglinid Hb subunit A1, A2, B1, B2 sequences. Red arrows indicate positions contain free cysteines or cysteine residues in HB B2 chains, and B1/A2 chains, respectively.

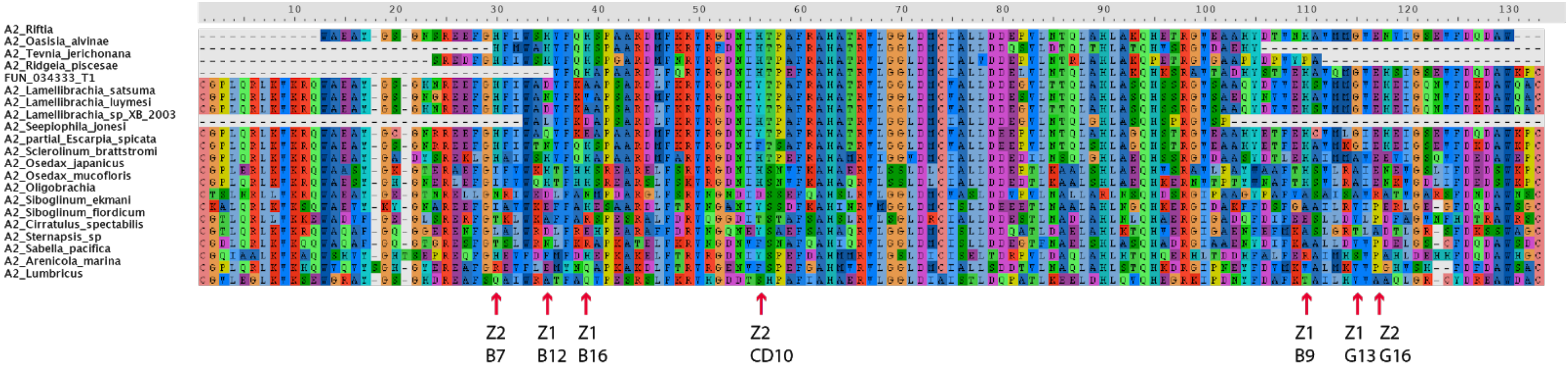

Figure S5. Partial alignment of sampled siboglinid HB subunit A2 sequences. Red arrows indicate amino acid residues at the interface between pairs of A2 chains with zinc moieties for H2S binding.

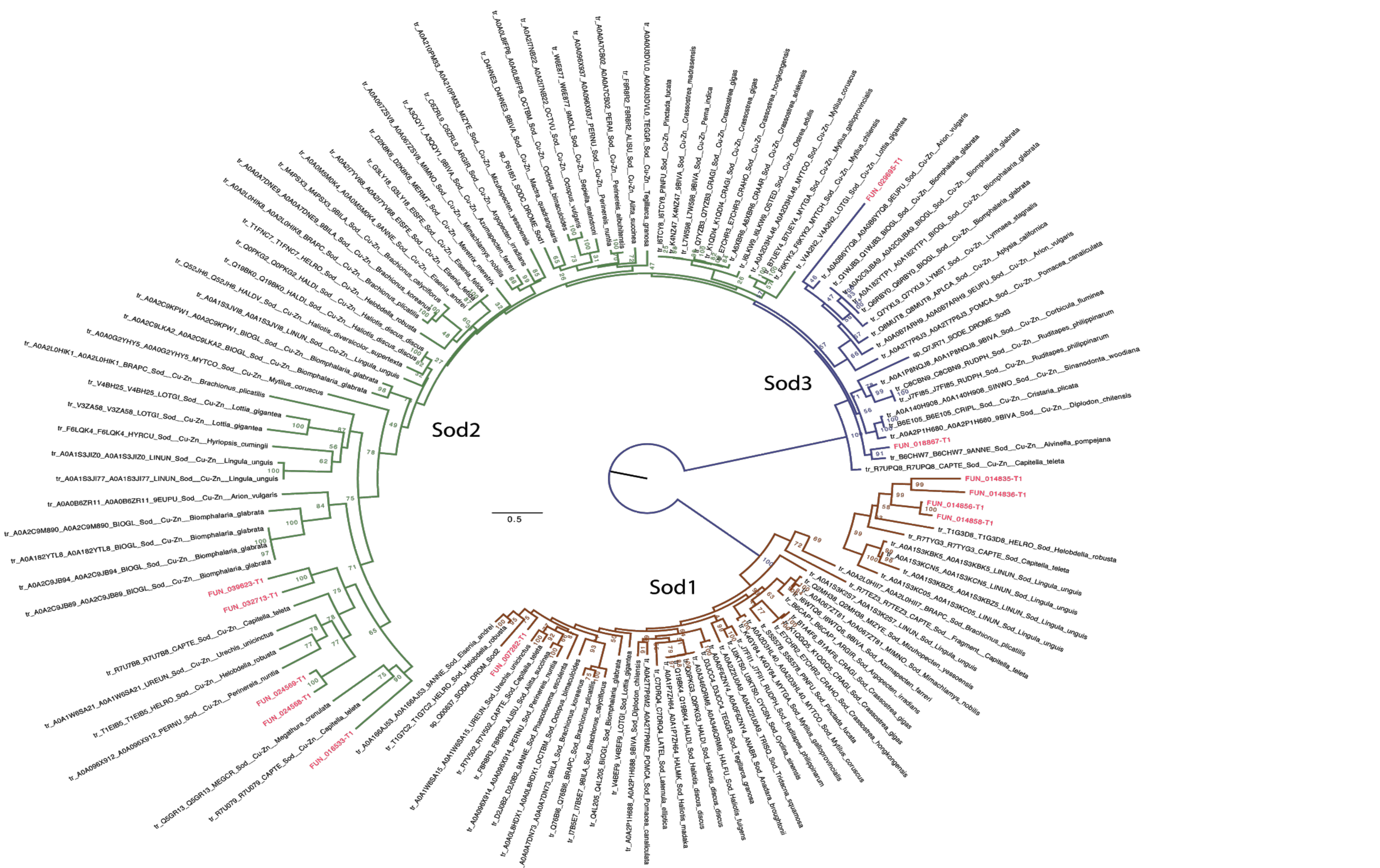

Figure S6. Lophotrochozoan SOD maximum-likelihood tree reconstructed with IQtree with midpoint rooting with 1000 ultrafast bootstraps using LG model. Bootstrap support values are shown at the relevant node. GenBank accession numbers are listed on the terminal nodes. GenBank accession numbers are next to the tip names.

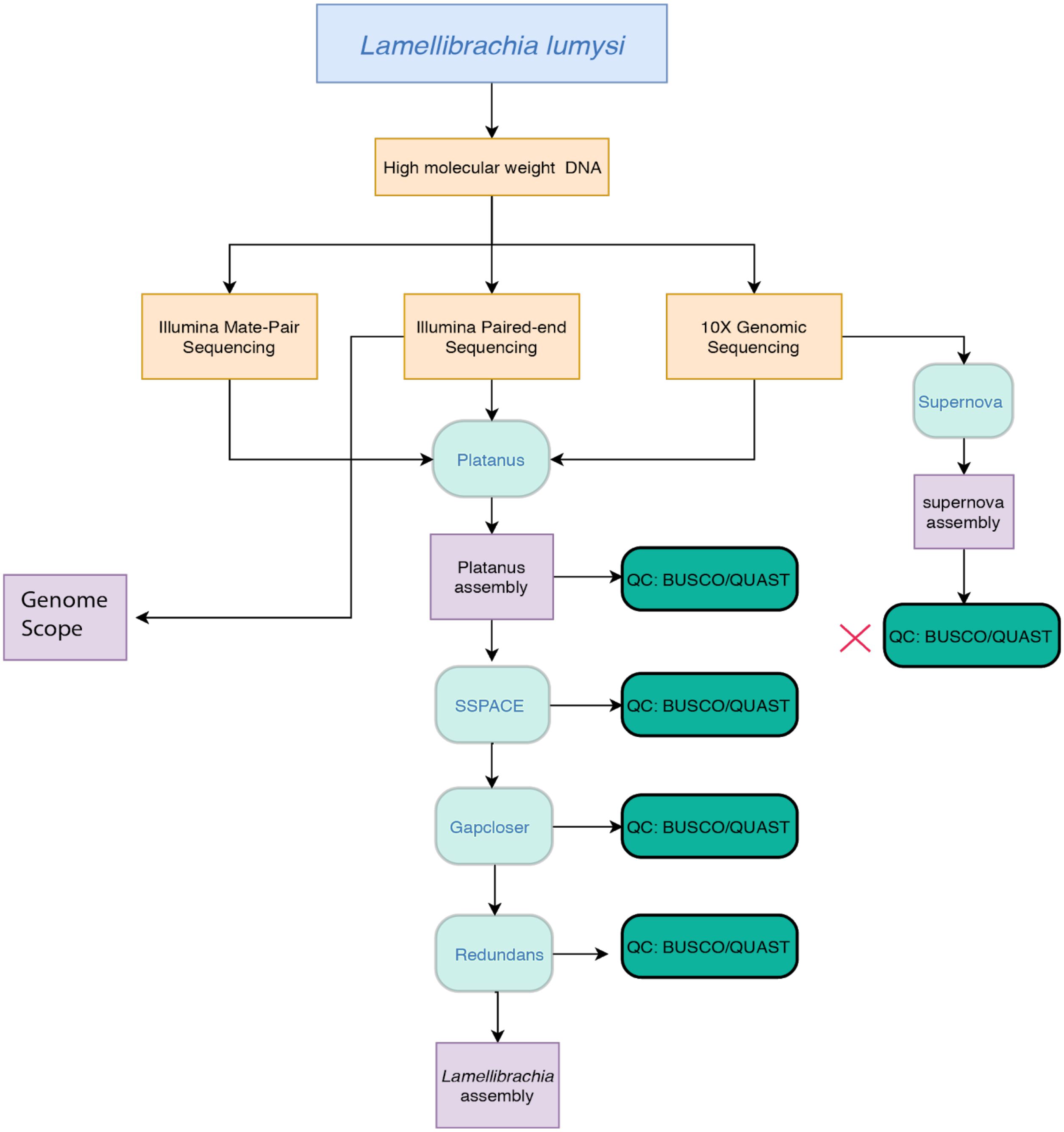

Figure S7. Workflow of *Lamellibrachia luymesi* genome assembly. 10X genomics assembly alone provide worse assembly and failed QC compared to Platanus indicated by red cross.

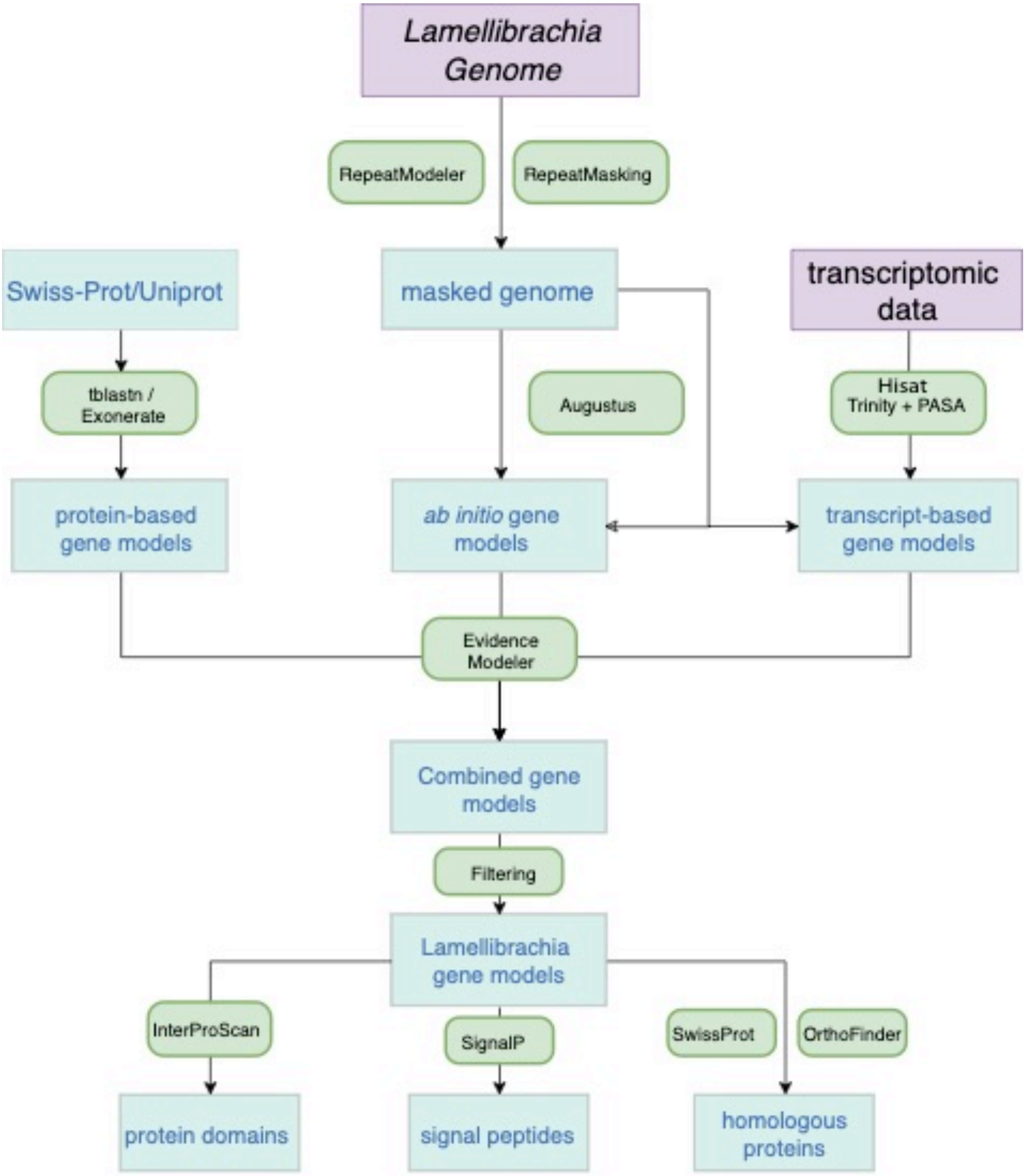

Figure S8. Workflow of *Lamellibrachia* genome annotation pipeline using Funannotate.

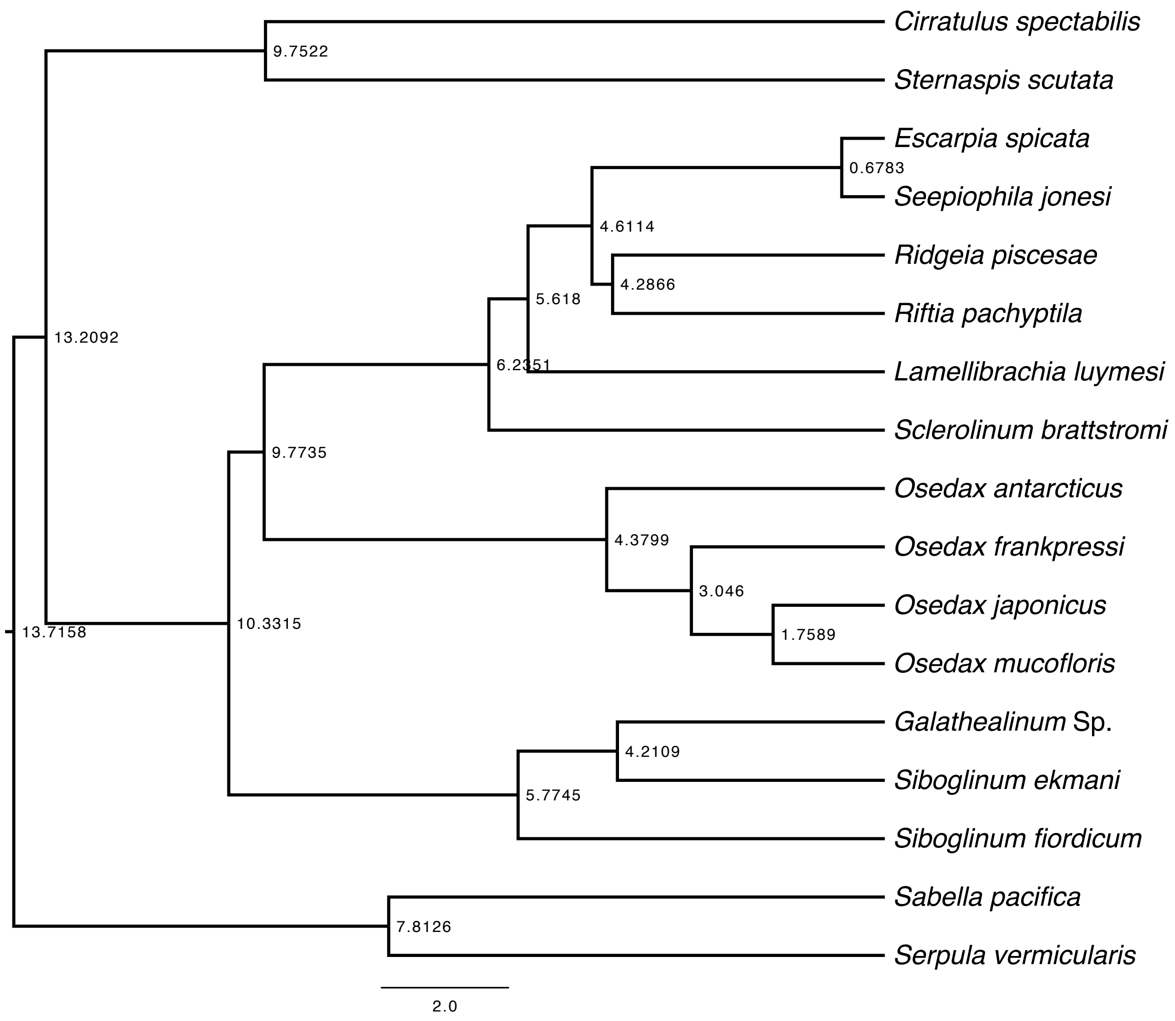

Figure S9. Uncalibrated phylogeny of Siboglinidae inferred with BEAST2 based on 191 OGs. Nodal support represents the nodal heights.

Table S1. Sequencing information of *Lamellibrachia* genome.

Tissue	Data Type	Sequencing Chemistry	Total Read Number	Lab Accession	Accession Number	Coverage (X)
**Vestimentum**	Genomics	10X Genomics	648,546,716	KH-4260-0006	SRR8519110	141.48
**Vestimentum**	Genomics	180 bp Paired-end	530,601,282	SL84794	SRR8519115	96.43
**Vestimentum**	Genomics	180 bp Paired-end	318,356,186	SL115013	SRR8519114	57.86
**Vestimentum**	Genomics	400 bp Paired-end	237,879,494	SL84795	SRR8519113	43.12
**Vestimentum**	Genomics	750 bp Paired-end	118,008,914	SL84796	SRR8519112	21.40
**Vestimentum**	Genomics	3-5 kbp Mate-pair	344,803,888	SL85812	SRR8519119	60.77
**Vestimentum**	Genomics	5-7 kbp Mate-pair	352,639,094	SL85813	SRR8519118	64.04
**Plume**	Transcriptome	Paired-end	58,660,044	SL85796	SRR8519117	11.31
**Trophosome**	Transcriptome	Paired-end	75,640,660	SL85798	SRR8519111	14.60
**Vestimentum**	Transcriptome	Paired-end	50,537,812	SL85797	SRR8519116	9.75
**Total**			2,735,674,090			520.76

Table S2.
Genome assembly and BUSCO statistics of *Lamellibrachia luymesi* compared to other lophotrochozoan genomes.

Taxon	Species Name	# contigs	Total length	Largest contig	GC (%)	N50	BUSCO (%)				
							Complete	Single	Duplicate	Fragment	Missing
**Annelida**	*Lamellibrachia lumysi*	11,871	687,711,696	2,117,112	40.16	372,990	95.80	93.00	2.80	2.90	1.30
	Capitella teleta	20,803	333,283,208	1,620,044	40.36	188,402	92.30	87.60	4.70	1.10	6.60
	Hydroides elegans	188,407	1,026,046,400	244,066	35.43	17,725	79.90	53.00	26.90	8.80	11.30
	Helobdella robusta	1,991	235,376,169	13,640,604	32.82	3,060,193	85.30	83.80	1.50	3.70	11.00
**Phoronida**	*Phoronis australis*	3,983	498,443,662	4,871,659	39.34	655,058	91.90	89.40	2.50	1.30	6.80
**Nemertea**	*Notospermus geniculatus*	11,108	858,599,399	1,576,180	42.85	239,235	91.90	89.40	2.50	1.30	6.80
**Mollusca**	*Crassostrea virginica*	10	684,723,884	104,168,038	34.83	75,944,018	90.70	88.10	2.60	0.80	8.50
	Crassostrea gigas	7,658	557,717,710	1,964,558	33.42	402,213	90.80	85.70	5.10	0.90	8.30
	Bathymodiolus platifrons	65,662	1,658,191,953	2,790,175	34.17	345,477	89.30	88.00	1.30	2.20	8.50
	Mytilus galloprovincialis	1,002,334	1,500,149,602	67,529	31.77	3,239	91.20	63.00	28.20	0.90	7.90
	Octopus bimaculoides	151,674	2,338,188,782	4,064,693	36.06	485,615	85.80	85.30	0.50	3.40	10.80
	Modiolus philippinarum	74,573	2,629,556,424	715,382	33.96	100,386	85.20	82.70	2.50	4.90	9.90
	Mizuhopecten yessoensis	82,658	987,568,220	7,498,238	36.52	827,226	89.80	87.80	2.00	1.20	9.00
	Lottia gigantea	4,469	359,505,668	9,386,848	33.28	1,870,055	91.30	90.20	1.10	0.90	7.80
	Aplysia californica	4,331	927,296,314	6,102,535	40.35	917,541	88.30	87.80	0.50	1.60	10.10
**Brachiopoda**	*Lingula anatina*	2,677	406,282,338	2,166,018	36.42	460,090	90.20	70.20	20.00	0.90	8.90

Table S3.

Proteomics and genome assemblies used in comparative analyses.

Taxon	Species	Genome source	RefSeq assembly accession
**Annelida**	*Lamellibrachia luymsi*	This study	SDWI00000000
	Capitella teleta	NCBI	GCA_000328365.1
	Helobdella robusta	NCBI	GCA_000326865.1
**Mollusca**	*Lottia gigantea*	NCBI	GCA_000327385.1
	Octopus bimaculoides	NCBI	GCA_001194135.1
	Chlamys farreri	Webserver	http://mgb.ouc.edu.cn/cfbase/html/
	Bathymodiolus platifrons	NCBI	GCA_002080005.1
	Biomphalaria glabrata	NCBI	GCF_000457365.1
	Mizuhopecten yessoensis	NCBI	GCA_002113885.2
	Modiolus philippinarum	NCBI	GCA_000457365.1
	Crassostrea gigas	NCBI	GCF_000297895.1
	Crassostrea virginica	NCBI	GCA_002022765.4
	Aplysia californica	NCBI	GCA_000002075.2
**Nemertea**	*Notospermus geniculatus*	NCBI	GCA_002633025.1
**Phoronida**	*Phoronis australis*	NCBI	GCA_002633005.1
**Brachiopoda**	*Lingula anatina*	NCBI	GCA_001039355.2
**Flatworm**	*Schistosoma mansoni*	NCBI	GCA_000237925.2
	Schmidtea mediterranea	NCBI	GCA_002600895.1
	Macrostomum lignano	NCBI	GCA_002269645.1
	Echinococcus multilocularis	NCBI	GCA_000469725.3
**Ecdysozoa**	*Diphania pulex*	NCBI	GCA_000187875.1
	Drosophila melanogaster	NCBI	GCA_000001215.4

Table S4.
Repetitive element contained in the *Lamellibrachia luymesi* genome

	Subclass	Number of elements	length occupied (bp)	percentage of sequence
**SINEs**		56,783	8,181,892	1.19
**LINEs**		307,497	96,235,311	13.99
	LINE1	1,333	337,473	0.05
	LINE2	51,791	18,267,908	2.66
	L3/CR1	83,528	33,202,610	4.83
**LTR elements**		70,108	17,360,126	2.52
**DNA elements**		442,010	101,825,130	14.81
	hAT-Charlie	6,246	1,710,664	0.25
	TcMar-Tigger	4,078	1,496,397	0.22
**Unclassified**		95,876	17,185,149	2.5
**Tot. interspersed repeats**			240,787,608	35.01
**Small RNA**		101	27,655	0
**Satellites**		813	376,172	0.05
**Simple repeats**		211,949	13,781,625	2
**Low complexity**		8,125	682,495	0.1

Table S5.
PANTHER gene family annotation of gene families that are under expansion or contraction as identified by CAFE.

Orthology Group	Number of gain and loss	PANTHER gene family	PANTHER annotation
**OG0000040**	+23*	PTHR44025	LOW-DENSITY LIPOPROTEIN RECEPTOR-RELATED PROTEIN
**OG0000044**	+16*	PTHR12011	G-PROTEIN COUPLED RECEPTOR
**OG0000059**	+25*	PTHR11177	CHITINASE
**OG0000062**	+20*	PTHR28576	PIGGYBAC TRANSPOSABLE ELEMENT-DERIVED PROTEIN
**OG0000075**	+20*	PTHR19325	COMPLEMENT COMPONENT-RELATED SUSHI DOMAIN-CONTAINING
**OG0000091**	+27*	PTHR11119	XANTHINE-URACIL / VITAMIN C PERMEASE FAMILY MEMBER
**OG0000118**	+30*	PTHR10877	POLYCYSTIN-RELATED
**OG0000124**	+9*	PTHR13802	MUCIN 4-RELATED
**OG0000128**	+23*	PTHR10131	TNF RECEPTOR ASSOCIATED FACTOR
**OG0000137**	+14*	PTHR11908	XANTHINE DEHYDROGENASE
**OG0000146**	+18*	PTHR11709	MULTI-COPPER OXIDASE
**OG0000150**	+20*	PTHR14647	GALACTOSE-3-O-SULFOTRANSFERASE
**OG0000155**	+13*	PTHR23033	BETA1,3-GALACTOSYLTRANSFERASE
**OG0000171**	+17*	PTHR10283	SOLUTE CARRIER FAMILY 13 MEMBER
**OG0000180**	+16*	PTHR24039	FIBRILLIN
**OG0000183**	+21*	PTHR14453	PARP/ZINC FINGER CCCH TYPE DOMAIN CONTAINING PROTEIN
**OG0000184**	+35*	PTHR24221	ABC TRANSPORTER
**OG0000198**	+27*	PTHR24033	FAMILY NOT NAMED
**OG0000219**	+24*	PTHR10579	CALCIUM-ACTIVATED CHLORIDE CHANNEL REGULATOR
**OG0000238**	+26*	PTHR11039	NEBULIN
**OG0000242**	+11*	PTHR34415	FAMILY NOT NAMED
**OG0000247**	+15*	PTHR14453	PARP/ZINC FINGER CCCH TYPE DOMAIN CONTAINING PROTEIN
**OG0000250**	+29*	PTHR13800	TRANSIENT RECEPTOR POTENTIAL CATION CHANNEL, SUBFAMILY M, MEMBER 6
**OG0000262**	+16*	PTHR43645	UPF0214 PROTEIN YFEW
**OG0000268**	+13*	PTHR43998	FILAMIN
**OG0000277**	+13*	PTHR44131	CUB DOMAIN-CONTAINING PROTEIN
**OG0000287**	+18*	PTHR44097	PROTEIN SERRATE
**OG0000290**	+7*	PTHR10166	VOLTAGE-DEPENDENT CALCIUM CHANNEL SUBUNIT ALPHA-2/DELTA-RELATED
**OG0000293**	+26*	PTHR22605	AAA+ ATPASE, CORE DOMAIN-CONTAINING PROTEIN
**OG0000304**	+12*	PTHR23097	TUMOR NECROSIS FACTOR RECEPTOR SUPERFAMILY MEMBER
**OG0000305**	+17*	PTHR23232	KRAB DOMAIN C2H2 ZINC FINGER
**OG0000307**	+10*	PTHR18966	IONOTROPIC GLUTAMATE RECEPTOR
**OG0000318**	+11*	PTHR12622	DELTEX-RELATED
**OG0000322**	+17*	PTHR23024	MEMBER OF 'GDXG' FAMILY OF LIPOLYTIC ENZYMES
**OG0000324**	+8*	PTHR11106	GANGLIOSIDE INDUCED DIFFERENTIATION ASSOCIATED PROTEIN 2-RELATED

**OG0000337**	+24*	PTHR10887	DNA2/NAM7 HELICASE FAMILY
**OG0000342**	+25*	PTHR16897	CARNOSINE N-METHYLTRANSFERASE
**OG0000347**	+11*	PTHR10796	PATCHED-RELATED
**OG0000357**	+10*	PTHR23302	TRANSMEMBRANE CHANNEL-RELATED
**OG0000365**	+7*	PTHR12042	LACTOSYLCERAMIDE 4-ALPHA-GALACTOSYLTRANSFERASE ALPHA- 1,4-GALACTOSYLTRANSFERASE
**OG0000373**	+23*	PTHR31649	LD46221P-RELATED
**OG0000378**	+39*	PTHR13715	RYANODINE RECEPTOR AND IP3 RECEPTOR
**OG0000388**	+16*	PTHR15600	SACSIN
**OG0000398**	+13*	PTHR11697	GENERAL TRANSCRIPTION FACTOR 2-RELATED ZINC FINGER PROTEIN
**OG0000423**	+8*	PTHR44014	FAMILY NOT NAMED
**OG0000432**	+14*	PTHR14454	GRB2-ASSOCIATED AND REGULATOR OF MAPK PROTEIN
**OG0000460**	+10*	PTHR43905	CONTACTIN
**OG0000465**	+11*	PTHR23130	FERRIC-CHELATE REDUCTASE
**OG0000468**	+15*	PTHR16897	CARNOSINE N-METHYLTRANSFERASE
**OG0000477**	+10*	PTHR11616	SODIUM/CHLORIDE DEPENDENT TRANSPORTER
**OG0000491**	+20*	PTHR11046	OLIGORIBONUCLEASE, MITOCHONDRIAL
**OG0000500**	+12*	PTHR24106	CASPASE RECRUITMENT DOMAIN-CONTAINING PROTEIN 8/NACHT, LRR AND PYD DOMAINS-CONTAINING PROTEIN
**OG0000524**	+50*	PTHR19277	PENTRAXIN
**OG0000555**	+9*	PTHR31009	S-ADENOSYL-L-METHIONINE:CARBOXYL METHYLTRANSFERASE FAMILY PROTEIN
**OG0000562**	+8*	PTHR15698	PHYTANOYL-COA HYDROXYLASE-INTERACTING PROTEIN
**OG0000570**	+27*	PTHR10697	MAMMALIAN EPENDYMIN-RELATED PROTEIN 1
**OG0000578**	+21*	PTHR33748	FAMILY NOT NAMED
**OG0000586**	+14*	PTHR34153	SI:CH211-262H13.3
**OG0000592**	+41*	PTHR19325	COMPLEMENT COMPONENT-RELATED SUSHI DOMAIN-CONTAINING
**OG0000613**	+11*	PTHR44854	FIBROCYSTIN-
**OG0000617**	+20*	PTHR19325	COMPLEMENT COMPONENT-RELATED SUSHI DOMAIN-CONTAINING
**OG0000640**	+28*	PTHR23145	NUCLEOSOMAL BINDING PROTEIN 1
**OG0000656**	+9*	PTHR19297	GLYCOSYLTRANSFERASE 14 FAMILY MEMBER
**OG0000687**	+13*	PTHR24243	G-PROTEIN COUPLED RECEPTOR
**OG0000715**	+10*	PTHR10796	PATCHED-RELATED
**OG0000728**	+22*	PTHR45240	ZINC METALLOPROTEINASE NAS
**OG0000789**	+14*	PTHR10773	DNA-DIRECTED RNA POLYMERASES I, II, AND III SUBUNIT RPABC2
**OG0000849**	+37*	PTHR12673	FACIOGENITAL DYSPLASIA PROTEIN
**OG0000862**	+23*	PTHR11514	MYC
**OG0000917**	+10*	PTHR31569	ZINC FINGER SWIM DOMAIN-CONTAINING PROTEIN
**OG0000927**	+21*	PTHR44252	CARBONYL REDUCTASE NADPH
**OG0000938**	+17*	PTHR11360	MONOCARBOXYLATE TRANSPORTER
**OG0000943**	+39*	PTHR13954	IRE1-RELATED
**OG0000987**	+10*	PTHR24280	CYTOCHROME P450 20A1

**OG0001012**	+19*	PTHR16897	CARNOSINE N-METHYLTRANSFERASE
**OG0001096**	+11*	PTHR11799	PARAOXONASE
**OG0001166**	+10*	PTHR16897	HISTAMINE N-METHYLTRANSFERASE
**OG0001243**	+12*	PTHR11903	PROSTAGLANDIN G/H SYNTHASE
**OG0001293**	+52*	PTHR23194	PYGOPUS
**OG0001377**	+10*	PTHR31513	GLYCINE-RICH PROTEIN
**OG0001383**	+9*	PTHR20956	HEH2P
**OG0001422**	+41*	PTHR23259	RIDDLE
**OG0001533**	+24*	PTHR37558	FAMILY NOT NAMED
**OG0001594**	+10*	PTHR37445	FAMILY NOT NAMED
**OG0001671**	+22*	PTHR10199	THROMBOSPONDIN
**OG0001730**	+7*	PTHR24106	NACHT, LRR AND PYD DOMAINS-CONTAINING PROTEIN
**OG0001853**	+6*	PTHR13627	FUKUTIN RELATED PROTEIN
**OG0001862**	+10*	PTHR11697	GENERAL TRANSCRIPTION FACTOR 2-RELATED ZINC FINGER PROTEIN
**OG0001954**	+10*	PTHR15031	CARTILAGE INTERMEDIATE LAYER PROTEIN CLIP
**OG0002014**	+17*	PTHR12419	OTU DOMAIN CONTAINING PROTEIN
**OG0002286**	+6*	NONE	NA
**OG0002380**	+6*	NONE	NA
**OG0002381**	+22*	PTHR11046	OLIGORIBONUCLEASE, MITOCHONDRIAL
**OG0002455**	+11*	PTHR35558	FAMILY NOT NAMED
**OG0000077**	-10*	PTHR14918	PROTEIN SZT2

Table S6.
Genes under positive selection in *L. luymesi* genome.

OG	Gene	P-value	Gene Function	Gene abbreviation
**OG0006957**	LLUY_000212-T1	0.02302	Spliceosome-associated protein 49	SF3B4
**OG0004340**	LLUY_001076-T1	0.04523	Alpha-actinin, sarcomeric	
**OG0006468**	LLUY_001334-T1	0.01713	Calnexin	
**OG0006928**	LLUY_001589-T1	0.01382	Elongation factor Ts, mitochondrial	
**OG0004897**	LLUY_002297-T1	0.01114	Poly(U)-binding-splicing factor half pint	
**OG0008548**	LLUY_002984-T1	0.00546	Dynactin subunit 4	DCTN4
**OG0006763**	LLUY_003619-T1	0.00709	Cyclin-Y	
**OG0004056**	LLUY_004631-T1	0.01613	Catenin beta	ARM
**OG0002524**	LLUY_005084-T1	0.00311	Solute carrier	SLC35C2
**OG0003102**	LLUY_005263-T1	0.03916	Flotillin	LOC106174607
**OG0006666**	LLUY_005506-T1	0.02273	SWI/SNF-related matrix-associated actin-dependent regulator of chromatin subfamily A member 5	SMARCA5
**OG0007864**	LLUY_009597-T1	0.00909	Ubiquitin like with PHD and ring finger domains 1	UHRF1
**OG0004908**	LLUY_009843-T1	0.00392	Abhydrolase domain-containing protein 16A	ABHD16A
**OG0003380**	LLUY_010222-T1	0.00735	Cytoplasmic aconitate hydratase	ACO1
**OG0007468**	LLUY_010470-T1	0.00848	Mitochondria associated granulocyte macrophage csf signaling molecule	
**OG0002854**	LLUY_010487-T1	0.00986	Polyadenylate-binding protein 4 isoform 3	
**OG0001374**	LLUY_011873-T1	0.00001	Heterogeneous nuclear ribonucleoprotein K isoform a	
**OG0007217**	LLUY_012584-T1	0.00563	DEAD (Asp-Glu-Ala-Asp) box helicase 17	
**OG0003091**	LLUY_012601-T1	0.04956	NAD-dependent malate dehydrogenase	
**OG0008840**	LLUY_013348-T1	0.04147	DNA-directed RNA polymerase subunit	POLR3K
**OG0003281**	LLUY_015442-T1	0.00786	Histidyl-tRNA synthetase	HARS
**OG0003913**	LLUY_015497-T1	0.04182	Mitochondrial 2-oxoglutarate/malate carrier protein isoform 1	SLC25A11
**OG0002743**	LLUY_017472-T1	0.03782	AP complex subunit sigma	
**OG0000833**	LLUY_017711-T1	0.03678	Tropomyosin	
**OG0009436**	LLUY_017726-T1	0.02125	DTW domain-containing protein 2	DTWD2
**OG0009320**	LLUY_017796-T1	0.03177	Eukaryotic translation initiation factor 3 subunit E	EIF3E
**OG0005746**	LLUY_018903-T1	0.0353	Eukaryotic translation initiation factor 3 subunit G-B	EIF3G
**OG0003795**	LLUY_019377-T1	0.00004	Transformer-2 protein like protein beta	TRA2A
**OG0005969**	LLUY_019453-T1	0.00643	WW domain-binding protein 11	WBP11
**OG0008801**	LLUY_019831-T1	0.01402	Ubiquitinyl hydrolase 1	USP33
**OG0006721**	LLUY_019880-T1	0.04033	apoptogenic protein 1, mitochondrial isoform X2	
**OG0003274**	LLUY_020067-T1	0.0307	RAP1B, member of RAS oncogene family	RAP1B
**OG0005725**	LLUY_020293-T1	0.03946	Vang-like protein 1 isoform 1	VANGL1
**OG0002652**	LLUY_020307-T1	0.02661	Ets transcription factor	
**OG0006478**	LLUY_021145-T1	0.00467	Serine/threonine protein kinase gpbp	COL4A3BP
**OG0008049**	LLUY_021249-T1	0.0003	Spectrin alpha chain	
**OG0005771**	LLUY_021463-T1	0.01321	Cyclin-dependent kinase 11B	

**OG0008637**	LLUY_022209-T1	0.00786	Fumarylacetoacetate hydrolase domain-containing protein 2A	
**OG0005900**	LLUY_022472-T1	0.00455	Small subunit ribosomal protein S25e	RPS25
**OG0007287**	LLUY_022652-T1	0.00593	G protein subunit alpha q	GNA11
**OG0008914**	LLUY_023217-T1	0.04455	Iron-sulfur cluster assembly enzyme mitochondrial	
**OG0005815**	LLUY_025859-T1	0.02147	Eukaryotic translation initiation factor 3 subunit K	EIF3K
**OG0007368**	LLUY_026739-T1	0.00952	Methylmalonate-semialdehyde dehydrogenase	
**OG0005888**	LLUY_026882-T1	0.04049	Phosphoglycerate mutase	GPMA
**OG0000785**	LLUY_026914-T1	0.01486	Hexosyltransferase	
**OG0006908**	LLUY_027394-T1	0.00645	Vacuolar-sorting protein SNF8	SNF8
**OG0004910**	LLUY_028224-T1	0.03574	Calcium-transporting ATPase	
**OG0009027**	LLUY_028356-T1	0.0301	Putative molecular chaperone dnaj superfamily	
**OG0000154**	LLUY_029764-T1	0.00011	Glutathione-S-transferase	
**OG0009346**	LLUY_029786-T1	0.00846	Mitogen-activated protein kinase kinase kinase 7-interacting protein 1	TAB1
**OG0004057**	LLUY_030290-T1	0.02791	Putative cytochrome b5 ixodes scapularis cytochrome b5 ixodes pacificus cytochrome b5	
**OG0003870**	LLUY_032842-T1	0.00054	LOW QUALITY PROTEIN: cyclin-L1	
**OG0006261**	LLUY_034019-T1	0.02227	USO1 vesicle transport factor	USO1
**OG0004296**	LLUY_035988-T1	0.02737	Nuclear pore glycoprotein p62	
**OG0008323**	LLUY_036278-T1	0.039	Adenylate kinase	
**OG0003652**	LLUY_037138-T1	0.03401	3-methylcrotonyl-CoA carboxylase (Fragment)	
**OG0007377**	LLUY_037566-T1	0.00792	Succinate dehydrogenase [ubiquinone] flavoprotein subunit, mitochondrial	
**OG0004872**	LLUY_038712-T1	0.03539	RING finger protein 157	
**OG0005567**	LLUY_038872-T1	0.0268	Beclin 1 (Coiled-coil, myosin-like BCL2 interacting protein)	
**OG0008960**	LLUY_040090-T1	0.03399	Proteasome endopeptidase complex	
**OG0008006**	LLUY_040359-T1	0.01129	Pre-mRNA-processing-splicing factor	
**OG0000226**	LLUY_031362-T1	0.01356		
**OG0000301**	LLUY_024040-T1	0.03886		
**OG0000415**	LLUY_012131-T1	0.04791		
**OG0000553**	LLUY_017958-T1	0.03006		
**OG0000699**	LLUY_004437-T1	0.02501		
**OG0000748**	LLUY_001501-T1	0.00247		
**OG0001380**	LLUY_009633-T1	0.04654		
**OG0001834**	LLUY_021867-T1	0.00191		
**OG0001853**	LLUY_004480-T1	0.00041		
**OG0002737**	LLUY_026832-T1	0.03011		
**OG0002879**	LLUY_000452-T1	0		
**OG0003058**	LLUY_002435-T1	0.00369		
**OG0003244**	LLUY_017894-T1	0.0151		
**OG0003461**	LLUY_014892-T1	0.002		
**OG0003613**	LLUY_020571-T1	0.01552		

**OG0003673**	LLUY_020718-T1	0.00345		
**OG0003920**	LLUY_000944-T1	0.00525		
**OG0004443**	LLUY_033704-T1	0.01068		
**OG0004607**	LLUY_033929-T1	0.04947		
**OG0004661**	LLUY_017962-T1	0.00699		
**OG0005048**	LLUY_029701-T1	0.02074		
**OG0006133**	LLUY_015524-T1	0.02559		
**OG0006180**	LLUY_010981-T1	0.01042		
**OG0007484**	LLUY_035904-T1	0.00307		
**OG0007714**	LLUY_014959-T1	0.00013		
**OG0007953**	LLUY_008037-T1	0.02174		
**OG0008648**	LLUY_003324-T1	0.03275		
**OG0009134**	LLUY_007970-T1	0.02872		
**OG0009370**	LLUY_011377-T1	0.0085		

Table S7.

Key genes of host genes identified as proteins from proteomic analysis.

LLUYction System	Feature ID	LLUYction
**Poteosoma**	LLUY_028786-T1	PSMA6; 20S proteasome subunit alpha 1 [EC:3.4.25.1]
	LLUY_012964-T1	PSMA2; 20S proteasome subunit alpha 2 [EC:3.4.25.1]
	LLUY_008627-T1	PSMA4; 20S proteasome subunit alpha 3 [EC:3.4.25.1]
	LLUY_027290-T1	PSMA7; 20S proteasome subunit alpha 4 [EC:3.4.25.1]
	LLUY_003537-T1	PSMA5; 20S proteasome subunit alpha 5 [EC:3.4.25.1]
	LLUY_040090-T1	PSMA1; 20S proteasome subunit alpha 6 [EC:3.4.25.1]
	LLUY_012936-T1	PSMA3; 20S proteasome subunit alpha 7 [EC:3.4.25.1]
	LLUY_023805-T1	PSMB3; 20S proteasome subunit beta 1
	LLUY_029937-T1	PSMB3; 20S proteasome subunit beta 3
	LLUY_002855-T1	PSMB2; 20S proteasome subunit beta 4
	LLUY_015520-T1	PSMB5; 20S proteasome subunit beta 5
	LLUY_022140-T1	PSMB1; 20S proteasome subunit beta 6
	LLUY_022807-T1	PSMB4; 20S proteasome subunit beta 7
	LLUY_003098-T1	SMD2, RPN1; 26S proteasome regulatory subunit N1
	LLUY_026382-T1	PSMD7, RPN8; 26S proteasome regulatory subunit N8
	LLUY_017796-T1	EIF3E, INT6; translation initiation factor 3 subunit E
	LLUY_014232-T1	HSPA1s; heat shock 70kDa protein 1/2/6/8
	LLUY_037621-T1	HSP90A, htpG; molecular chaperone HtpG
	LLUY_000099-T1	HSP90A, htpG; molecular chaperone HtpG
	LLUY_000099-T1	RAD23, HR23; UV excision repair protein RAD23
**Lysosome**	LLUY_033571-T1	cathepsin C [EC:3.4.14.1]
	LLUY_007735-T1, LLUY_007737-T1, LLUY_036642-T1, LLUY_036643-T1	cathepsin B [EC:3.4.22.1]
	LLUY_009810-T1, LLUY_026908-T1	cathepsin L [EC:3.4.22.15]
	LLUY_012982-T1	legumain [EC:3.4.22.34]
	LLUY_007693-T1	cathepsin F [EC:3.4.22.41]
	LLUY_016715-T1	cathepsin D [EC:3.4.23.5]
	LLUY_023349-T2, LLUY_023349-T1	clathrin heavy chain
	LLUY_017989-T1	lysosomal-associated membrane protein 1/2
	LLUY_024617-T1	cathepsin X [EC:3.4.18.1]
	LLUY_002319-T1	lysosomal alpha-mannosidase [EC:3.2.1.24]
	LLUY_004297-T1	lysosomal alpha-glucosidase [EC:3.2.1.20]
	LLUY_005359-T1	saposin
	LLUY_016480-T1	lysosome membrane protein 2
	LLUY_003890-T1	cathepsin A (carboxypeptidase C) [EC:3.4.16.5]
**Longevity regulating pathway**	LLUY_007262-T1, LLUY_014836-T1, LLUY_014856-T1	SOD2; superoxide dismutase, Fe-Mn family [EC:1.15.1.1]
	LLUY_018867-T1	SOD1; superoxide dismutase, Cu-Zn family [EC:1.15.1.1]

Table S8.

Key genes of symbiont genes identified as proteins from proteomic analysis.

Function System	Feature ID	Length (bp)	Function
**rTCA Cycle**	Lamellibrachia_symbiont.peg.150	1524	Fumarate hydratase class I, aerobic (EC 4.2.1.2)
	Lamellibrachia_symbiont.peg.2185	1911	2-oxoglutarate oxidoreductase, alpha subunit (EC 1.2.7.3)
	Lamellibrachia_symbiont.peg.2186	954	2-oxoglutarate oxidoreductase, beta subunit (EC 1.2.7.3)
	Lamellibrachia_symbiont.peg.2943	987	Malate dehydrogenase (EC 1.1.1.37)
	Lamellibrachia_symbiont.peg.986	1161	ATP citrate lyase beta chain (EC 4.3.1.8)
	Lamellibrachia_symbiont.peg.987	870	ATP citrate lyase alpha chain (EC 4.3.1.8)
	Lamellibrachia_symbiont.peg.2924	1167	2-oxoglutarate oxidoreductase, alpha subunit (EC 1.2.7.3)
	Lamellibrachia_symbiont.peg.2925	999	2-oxoglutarate oxidoreductase, beta subunit (EC 1.2.7.3)
	Lamellibrachia_symbiont.peg.2926	570	2-oxoglutarate oxidoreductase, gamma subunit (EC 1.2.7.3)
**Calvin Cycle**	Lamellibrachia_symbiont.peg.2754	1389	Ribulose bisphosphate carboxylase (EC 4.1.1.39)
	Lamellibrachia_symbiont.peg.2757	804	Rubisco activation protein CbbQ
	Lamellibrachia_symbiont.peg.2758	2256	Rubisco activation protein CbbO
**Sulfer Oxidation**	Lamellibrachia_symbiont.peg.303	942	Dissimilatory sulfite reductase, beta subunit (EC 1.8.99.3)
**Nitrogen Metabolism**	Lamellibrachia_symbiont.peg.724	1602	Respiratory nitrate reductase beta chain (EC 1.7.99.4Denitrifying reductase gene clusters; Nitrate and nitrite ammonification
	Lamellibrachia_symbiont.peg.725	3762	Respiratory nitrate reductase alpha chain (EC 1.7.99.4)
**Adhesion-related proteins**	Lamellibrachia_symbiont.peg.2820	978	Ankyrin
	Lamellibrachia_symbiont.peg.2856	3813	Fibronectin type III domain protein
**Oxidative stress**	Lamellibrachia_symbiont.peg.1649	582	Superoxide dismutase [Fe] (EC 1.15.1.1) (FeSOD)
	Lamellibrachia_symbiont.peg.2936	489	Rubrerythrin

Table S9.
Lamellibrachia Hb sequences identified that are highly expressed in the trophosome tissue or as proteins from proteomic data.

HBs	*Lamellibrachia luymsei* Hbs	Differential expressed	Mass spectrum
A1 Chain	LLUY_034331-T1	*	*
	LLUY_034332-T1	*	*
A2 Chain	LLUY_034333-T1	*	*
B2 Chain	LLUY_029258-T1	x	*
B1 Chain	LLUY_004752-T1	*	*
	LLUY_004753-T1	*	x
	LLUY_005026-T1	x	x
	LLUY_005027-T1	x	x
	LLUY_005030-T1	*	x
	LLUY_005031-T1	x	x
	LLUY_009666-T1	x	x
	LLUY_009670-T1	x	x
	LLUY_009671-T1	*	x
	LLUY_009673-T1	x	x
	LLUY_013447-T1	*	*
	LLUY_013449-T1	*	x
	LLUY_017246-T1	*	*
	LLUY_017247-T1	*	*
	LLUY_020152-T1	*	x
	LLUY_026555-T1	x	x
	LLUY_026556-T1	*	*
	LLUY_029945-T1	x	x
	LLUY_032994-T1	x	x
	LLUY_038743-T1	x	x
	LLUY_039230-T1	x	x
	LLUY_040024-T1	x	x
	LLUY_004634-T1	*	x
Linkers	LLUY_002344-T1	*	*
	LLUY_024479-T1	*	x
	LLUY_026441-T1	*	*
	LLUY_026443-T1	*	*

*: Positive; x: negative.
Sequences with putative free-cystenine were colored as red.

Table S10.
Number of unique TLR proteins encoded in lophotrochozoan genomes.

Taxon	Species	Number of TLR identified	Number of RLR identified
**Annelida**	*Lamellibrachia luymesi*	33	2
	Capitella telata	5	3
	Helobdella robusta	4	3
**Mollusca**	*Bathymodiolus platifrons*	61	7
	Crassostrea gigas	61	11
	Modiolus philippinarum	90	2
	Mizuhopecten yessoensis	30	4
	Octopus bimaculoides	5	5
	Patinopecten yessoensis	22	4
	Lottia gigantea	7	3
	Crassostrea virginica	109	8
	Biomphalaria glabrata	17	6
	Aplysia californica	15	2
**Nemertea**	*Notospermus geniculatus*	5	4
**Phoronida**	*Phoronis australis*	23	3
**Brachiopoda**	*Lingula anatina*	46	13
**Vertebrata**	*Homo sapiens*	11	3

Table S11.

Taxon sampling and source of data used in molecular clock analyses.

Taxon	Clade	Data	Reads	Source	Accession #s
Riftia pachyptila	Siboglinidae - Vestimentifera	454	1,333,110	NCBI SRA	SRR346550
Riftia pachyptila	Siboglinidae - Vestimentifera	454	623,927	NCBI SRA	SRR346549
Escarpia spicata	Siboglinidae - Vestimentifera	454	283,594	NCBI SRA	SRR3554587
Lamellibrachia luymesi	Siboglinidae - Vestimentifera	Illumina		Genome	
Ridgeia piscesae	Siboglinidae - Vestimentifera	454	1,092,906	NCBI SRA	SRR346554
Ridgeia piscesae	Siboglinidae - Vestimentifera	Sanger	515	NCBI EST	EV802484 - EV802997, EV823675
Seepiophila jonesi	Siboglinidae - Vestimentifera	454	382,144	NCBI SRA	SRR3554599
Sclerolinum brattstromi	Siboglinidae - *Sclerolinum*	Illumina	44,207,372	NCBI SRA	SRR3560108
Osedax mucofloris	Siboglinidae - *Osedax*	Illumina	56,067,578	NCBI SRA	SRR3574511
Osedax rubiplumus	Siboglinidae - *Osedax*	Illumina	50,339,804	NCBI SRA	SRR3574382
Osedax frankpressi	Siboglinidae - *Osedax*	Illumina	137,706,423	NCBI SRA	SRX1024021
Osedax japonicus	Siboglinidae - *Osedax*	Illumina	36,774,348	NCBI SRA	DRX038901
Siboglinum fiordicum	Siboglinidae - Frenulata	Illumina	35,922,776	NCBI SRA	SRR3560206
Siboglinum ekmani	Siboglinidae - Frenulata	Illumina	63,511,320	NCBI SRA	SRR3560562
***Galathealinum* sp.**	Siboglinidae - Frenulata	454	456,440	NCBI SRA	SRX1842875
***Sternaspsis* sp.**	Sternaspidae	Illumina	54,186,104	NCBI SRA	SRR3574594
Cirratulus spectabilis	Cirratulidae	Illumina	57,767,330	NCBI SRA	SRR3574861

Table S12.
Domain requirements for identifying components of TLR pathway.

Protein	Domain Requirements
TLR/TOLL	TIR+LRR(≥3)
MYD88	TIR+DEATH
SARM1	TIR+SAM(2)
DDX58	CARD(2)+Helicase_ATP_binding+Helicase_C_Terminal+Rig1_Regulatory_Domain
DHX58	Helicase_ATP_binding+Helicase_C_Terminal+Rig1_Regulatory_Domain
IFIH1	CARD(2)+Helicase_ATP_binding+Helicase_C_Terminal+Rig1_Regulatory_Domain
TRAF	Zn_Finger+MATH/TRAF or Zn_Finger+WD40_repeats(≥3)
NLR	(x)+NACHT+LRR(≥3)
IKK	Kinase
IKB	ANK(≥3)
NFKB	RHD+ANK(≥3)+DEATH
NEMO	NEMO/Coiled_coil
IRF	Interferon_regulatroy_factor_DNA_binding_domain
