# Supplementary material for: Genomic adaptations to chemosymbiosis in the deep-sea seep-dwelling tubeworm Lamellibrachia luymesi
Source: BMC Biol. 2019 Nov 18;17:91. doi: 10.1186/s12915-019-0713-x (PMC6862839; doi:10.1186/s12915-019-0713-x)
Supplement: Supplementary file 3 — Additional file 3: Bioinformatic command lines used in this study. [file 12915_2019_713_MOESM3_ESM.pdf]

Genomic adaptations to chemosymbiosis in the deep-sea seep-dwelling tubeworm *Lamellibrachia luymesi*

Additional file 3: commands and parameters used in this study

1. Quality trimming of raw sequencing reads

1.1 paired-end libraries

Reads were trimmed using `trimmomatic-0.36` to remove low quality regions and adaptor for each paired-end library.

```
module load trimmomatic/0.36
java -jar /tools/trimmomatic-0.36/trimmomatic-0.36.jar PE -threads 20 -phred33  SL84795_1_1.fastq.gz  SL84795_1_2.fastq.gz SL84795_1_1.trim.R1.fq OUT.trim.unp1.fq SL84795_1_2.trim.R1.fq OUT.trim.unp2.fq ILLUMINACLIP:/tools/trimmomatic-0.36/adapters/TruSeq3-PE.fa:2:30:10 LEADING:3 TRAILING:3 SLIDINGWINDOW:4:15 MINLEN:36
java -jar /tools/trimmomatic-0.36/trimmomatic-0.36.jar PE -threads 20 -phred33  SL84795_2_1.fastq.gz  SL84795_2_2.fastq.gz SL84795_2_1.trim.R1.fq OUT.trim.unp1.fq SL84795_2_2.trim.R1.fq OUT.trim.unp2.fq ILLUMINACLIP:/tools/trimmomatic-0.36/adapters/TruSeq3-PE.fa:2:30:10 LEADING:3 TRAILING:3 SLIDINGWINDOW:4:15 MINLEN:36
java -jar /tools/trimmomatic-0.36/trimmomatic-0.36.jar PE -threads 20 -phred33  SL84796_1_1.fastq.gz  SL84796_1_2.fastq.gz SL84796_1_1.trim.R1.fq OUT.trim.unp1.fq SL84796_1_2.trim.R1.fq OUT.trim.unp2.fq ILLUMINACLIP:/tools/trimmomatic-0.36/adapters/TruSeq3-PE.fa:2:30:10 LEADING:3 TRAILING:3 SLIDINGWINDOW:4:15 MINLEN:36
java -jar /tools/trimmomatic-0.36/trimmomatic-0.36.jar PE -threads 20 -phred33  SL84796_2_1.fastq.gz  SL84796_2_2.fastq.gz SL84796_2_1.trim.R1.fq OUT.trim.unp1.fq SL84796_2_2.trim.R1.fq OUT.trim.unp2.fq ILLUMINACLIP:/tools/trimmomatic-0.36/adapters/TruSeq3-PE.fa:2:30:10 LEADING:3 TRAILING:3 SLIDINGWINDOW:4:15 MINLEN:36
```

1.2 Mate-pair libraries

Mate-pair reads were trimmed and sorted using `NxTrim v0.3.1` , and only “mp” (true mate-pair reads) and “unknown” (mostly large insert size reads) reads were used for downstream scaffolding analysis.

```
./nxtrim -1 ~/Lamellibrachia/mate/C62UMANXX_s5_1_7bp_Index_2_SL85812.fastq.gz  -2 ~/Lamellibrachia/mate/C62UMANXX_s5_2_7bp_Index_2_SL85812.fastq.gz -rf --separate -O Sl85812
./nxtrim -1 ~/Lamellibrachia/mate/C62UMANXX_s5_1_7bp_Index_2_SL85813.fastq.gz  -2 ~/Lamellibrachia/mate/C62UMANXX_s5_2_7bp_Index_2_SL85813.fastq.gz -rf --separate -O Sl85813
cat /scratch/yzl0084/clean/SL85812_mp_R2.fq ~/Lamellibrachia/SL85812_unknown_R2.fq >SL85812_nxtrim_R2.fq
cat /scratch/yzl0084/clean/SL85813_mp_R2.fq ~/Lamellibrachia/SL85813_unknown_R2.fq >SL85813_nxtrim_R2.fq
```

2. Genome assembly

Given high heterozygosity in non-model species, all reads were assembled using `Platanus v1.2.4`

```
~/bin/platanus assemble -k 31 -o Lamellibrachia_31 -f ./test/SL* ./SL8479[5-6]* -u 0.2 -d 0.3 -t 60 -m 1000
~/bin/platanus scaffold -o Lamellibrachia_31 -c Lamellibrachia_31_contig.fa -b Lamellibrachia_31_contigBubble.fa -IP1 ./test/SL84794.paired.A.fastq ./test SL84794.paired.B.fastq -IP2 ./SL84795.paired.A.fastq ./SL84795.paired.B.fastq -IP3 ./SL84796.paired.A.fastq ./SL84796.paired.B.fastq -OP4 /scratch/yzl0084/allpaths_assembly/Lamellibrachia/experiment/SL85812.trim.R1.fq /scratch/yzl0084/allpaths_assembly/Lamellibrachia/experiment/SL85812.trim.R2.fq  -OP5 /scratch/yzl0084/allpaths_assembly/Lamellibrachia/experiment/SL85813.trim.R1.fq /scratch/yzl0084/allpaths_assembly/Lamellibrachia/experiment SL85813.trim.R2.fq -u 0.2 -t 60
~/bin/platanus gap_close -o Lamellibrachia_31 -c Lamellibrachia_31_scaffold.fa  -IP1 ./test/SL84794.paired.A.fastq ./test/SL84794.paired.B.fastq -IP2 ./SL84795.paired.A.fastq ./SL84795.paired.B.fastq -IP3 ./SL84796.paire
d.A.fastq ./SL84796.paired.B.fastq -OP4 /scratch/yzl0084/allpaths_assembly/Lamellibrachia/experiment/SL85812.trim.R1.fq /scratch/yzl0084/allpaths_assembly/Lamellibrachia/experiment/SL85812.trim.R2.fq -OP5 /scratch/yzl0084/allpaths_assembly/Lamellibrachia/experiment/SL85813.trim.R1.fq /scratch/yzl0084/allpaths_assembly/Lamellibrachia/experiment/SL85813.trim.R2.fq -t 60
```

Gaps in scaffolds were filled with `GapCloser v1.12`.

```
GapCloser -b config -a Lamellibrachia.fa -o Lamellibrachia_gapclosed.fa -t 20
```

Redundant allele scaffolds were removed using `Redundans v0.13c`.

```
python2.7 ~/programs/redundans/redundans.py -v  --nogapclosing  --noscaffolding  -f  final_gapclosed.fa -o Lamellibrachia_redundant_final
```

Genome assembly quality was assessed with `QUAST v4.5`.

```
python ~/programs/quast-4.5/quast.py -o ark_quast -t 1 assembly.fa
```

Genome completeness with `BUSCO v3` using the Metazoa_odb9 database (978 Busco genes).

```
python2.7 ~/programs/busco/scripts/run_BUSCO.py -c 20 -o $FILENAME.out  -i $FILENAME -l ~/programs/busco/metazoa_odb9/ -m geno
```

3. Genome annotation

Gene models were constructed following the Funannotate pipeline 1.3.0 (https://github.com/nextgenusfs/funannotate (https://github.com/nextgenusfs/funannotate))

```
/home/yzl0084/programs/funannotate-master/funannotate train  --trinity  /scratch/yzl0084/Lamellibrachia/final/trinity.fasta -i /scratch/yzl0084/Lamellibrachia/MyAssembly.fa -l /scratch/yzl0084/Lamellibrachia/forward.fastq.gz -r /scratch/yzl0084/Lamellibrachia/reverse.fastq.gz --max_intronlen 14000  --stranded no --species "Lamellibrachia luymesi" --cpus 40 -o re_run
/home/yzl0084/programs/funannotate-master/funannotate predict --masked_genome /scratch/yzl0084/Lamellibrachia/final/genome.softmasked.fa --repeatmasker_gff3 /scratch/yzl0084/Lamellibrachia/final/repeatmasker.gff3 --exonerate_proteins /scratch/yzl0084/Lamellibrachia/final/exonerate.out \
        --transcript_evidence /scratch/yzl0084/Lamellibrachia/final/re_run/training/funannotate_train.trinity-GG.fasta \
        --rna_bam /scratch/yzl0084/Lamellibrachia/final/re_run/training/funannotate_train.coordSorted.bam \
        --pasa_gff /scratch/yzl0084/Lamellibrachia/final/re_run/training/funannotate_train.pasa.gff3 \
         -o /scratch/yzl0084/Lamellibrachia/final/re_run -s "Lamellibrachia luymsi" --cpus 40 --busco_db metazoa --organism other --max_intronlen 14000
#/home/yzl0084/programs/funannotate-master/funannotate update -i /scratch/yzl0084/Lamellibrachia/funannotate --cpus 30  -l /scratch/yzl0084/Lamellibrachia/forward.fastq.gz -r /scratch/yzl0084/Lamellibrachia/reverse.fastq.gz --stranded no
#/home/yzl0084/programs/funannotate-master/funannotate iprscan -i /scratch/yzl0084/Lamellibrachia/tubeworm -m local --iprscan_path /home/yzl0084/programs/interproscan-5.28-67.0/interproscan.sh -c 30 -o ipr
```

4. Orthology groups

Following all-to-all `Diamond v1.0`, `BLASTP` searches against 22 selected lophotrochozoan proteomes, orthology groups (OGs) were identified using Orthofinder with a default inflation parameter (I=1.5).

```
~/programs/OrthoFinder-2.2.1/orthofinder -f . -S  diamond -t 20 -M msa  -os
```

Gene families were annotated using `pantherscore` perl script.

```
 ~/programs/pantherScore2.1/pantherScore2.1.pl -l /tools/funannotate-1.5.0/db/PANTHER13.1/ -D B -V -i final.fa -o OG0000040.fa.tsv -n -s -c 20
```

Gene family expansion was followed the tutorial of `CAFE` (Here you can find the CAFE TUTORIAL: http://evomicsorg.wpengine.netdna-cdn.com/wp-content/uploads/2016/06/cafe_tutorial-1.pdf (http://evomicsorg.wpengine.netdna-cdn.com/wp-content/uploads/2016/06/cafe_tutorial-1.pdf)).

5. Manual annotation of gene families with potential interest

For gene family with particular interests, targeted genes were additionally processed through the `Extract_Homologs2` script used in (Tassia et al. 2017) (available at https://github.com/mtassia/Homolog_identification (https://github.com/mtassia/Homolog_identification)).

We used `MAFFT 7.2.15` to align Hb amino acid sequences.

```
for FILENAME in *.fasta; do mafft --auto --localpair --maxiterate 1000 $FILENAME > $FILENAME.aln; done
```

Maximum likelihood analyses were performed in `IQTree v1.5`.

```
iqtree -nt 8 -m [best-fit_model] -s [alignment_file] -pre [output_name]
```

6. Potential genes under positive selection.

We used `TranslaterX` to align nucleotide sequences of genes based on codon positions of each orthogroup.

```
for FILENAME in *.fasta; do translaterx.pl -p F -i $FILENAME -o $FILENAME -c 4 ; rm -rf *.aa_based_codon_coloured.html; rm -rf *nt[1-3]*; rm -rf *aaseqs*; rm -rf *.log; rm -rf *.html; done
```

Maximum likelihood analyses were performed in `IQTree v1.5`

```
iqtree -nt 8 -m [best-fit_model] -s [alignment_file] -pre [output_name]
```

Positive selection was calculated using the adaptive branch-site random effects likelihood (aBS-REL) model implemented in `HyPhy`.

7. Molecular clock of Siboglinidae.

Supermatrix dataset was constructed using `Agalma`.

```
agalma catalog insert --id OJAP  --paths OJAP_annotated_AA.fasta --species "Osedax japon
icus" --ncbi_id 385425
agalma catalog insert --id OFRA  --paths OFRA_annotated_AA.fasta --species "Osedax frank
pressi" --ncbi_id 283776
agalma catalog insert --id LLUY  --paths LLUY_annotated_AA.fasta --species "Lamellibrach
ia luymesi" --ncbi_id 238240
agalma catalog insert --id GSPP_annotated_AA.fasta  --paths GSPP_annotated_AA.fasta --sp
ecies "Galathealinum sp. YL-2016" --ncbi_id  1860144
agalma catalog insert --id CSPE_annotated_AA.fasta  --paths CSPE_annotated_AA.fasta --sp
ecies "Cirratulus spectabilis" --ncbi_id 307619
agalma catalog insert --id ESPI_annotated_AA.fasta  --paths ESPI_annotated_AA.fasta --sp
ecies "Escarpia spicata" --ncbi_id 53617
agalma catalog insert --id OART_annotated_AA.fasta  --paths OART_annotated_AA.fasta --sp
ecies "Osedax antarcticus" --ncbi_id 1369829
agalma catalog insert --id OMUC_annotated_AA.fasta  --paths OMUC_annotated_AA.fasta --sp
ecies "Osedax mucofloris" --ncbi_id 326170
agalma catalog insert --id RPAC_annotated_AA.fasta  --paths RPAC_annotated_AA.fasta --sp
ecies "Riftia pachyptila" --ncbi_id 6426
agalma catalog insert --id RPIS_annotated_AA.fasta  --paths RPIS_annotated_AA.fasta --sp
ecies "Ridgeia piscesae" --ncbi_id 27915
agalma catalog insert --id SBRA_annotated_AA.fasta  --paths SBRA_annotated_AA.fasta --sp
ecies "Sclerolinum brattstromi" --ncbi_id 167799
agalma catalog insert --id SEKM_annotated_AA.fasta  --paths SEKM_annotated_AA.fasta --sp
ecies "Siboglinum ekmani" --ncbi_id 167800
agalma catalog insert --id SFIO_annotated_AA.fasta  --paths SFIO_annotated_AA.fasta --sp
ecies "Siboglinum fiordicum" --ncbi_id 27908
agalma catalog insert --id SJON_annotated_AA.fasta  --paths SJON_annotated_AA.fasta --sp
ecies "Seepiophila jonesi" --ncbi_id 151527
agalma catalog insert --id SSPP_annotated_AA.fasta  --paths SSPP_annotated_AA.fasta --sp
ecies "Sternaspis scutata" --ncbi_id 36133
agalma catalog insert --id FSPP_annotated_AA.fasta  --paths FSPP_annotated_AA.fasta --sp
ecies "Nematostella vectensis" --ncbi_id 45351
agalma import --id OJAP --seq_type aa
agalma import --id OFRA --seq_type aa
agalma import --id LLUY --seq_type aa
agalma import --id GSPP --seq_type aa
agalma import --id CSPE --seq_type aa
agalma import --id ESPI --seq_type aa
agalma import --id OART --seq_type aa
agalma import --id OMUC --seq_type aa
agalma import --id RPAC --seq_type aa
agalma import --id RPIS --seq_type aa
agalma import --id SBRA --seq_type aa
agalma import --id SEKM --seq_type aa
agalma import --id SFIO --seq_type aa
agalma import --id SJON --seq_type aa
agalma import --id SSPP --seq_type aa
agalma import --id FSPP --seq_type aa
agalma -t 40 -m 100G homologize --id PhylogenyTest
```

Putative orthologous groups (OGs) were retrieved from each transcriptome following bioinformatics pipelines of Kocot et al. (2011) and Whelan et al. See Li et al. (2017) for more details (https://doi.org/10.1111/zsc.12201 (https://doi.org/10.1111/zsc.12201)).
